# Early public childcare and fertility: A longitudinal study for Europe

**DOI:** 10.1371/journal.pone.0353502

**Published:** 2026-07-14

**Authors:** Giovanni Minchio, Agnese Vitali, Stefani Scherer

**Affiliations:** Department of Sociology and Social Research, University of Trento, Trento, Italy; Gabriele d’Annunzio University of Chieti and Pescara: Universita degli Studi Gabriele d’Annunzio Chieti Pescara, ITALY

## Abstract

Empirical research reveals a considerable mismatch between desired and actual fertility across Europe, a mismatch which is more pronounced in countries characterized by limited policy support for families, suggesting that institutional interventions might be relevant in reducing this gap. This study analyzes the association between one of such policy interventions, namely public early childcare and education services (ECEC, age 0–2), and childbearing. The expectation is that an increase in the provision of public early childcare service in the region of residence will be positively associated with fertility. While existing evidence on whether childcare expansion increases fertility comes primarily from single-country studies or national-level comparisons, our empirical analysis combines sub-national information on regional public early childcare with longitudinal micro-level data from the European Union Statistics on Income and Living Conditions, from 2005 to 2020. Results show that an increase in regional ECEC is associated with higher probability of experiencing both transition to first and second birth and this association is significantly stronger in regions where ECEC was initially lower. Associations between childcare expansions and first birth transitions are particularly pronounced among lower-educated or employed women, while associations with second birth transitions are similar across all socio-economic groups, yet statistically non-significant among lower-educated mothers. Our results suggest that investing in public childcare is associated, in the short term, with reducing the gap between intended and actual fertility in Europe, particularly in contexts where provision is lower.

## Introduction

Over the past four decades, declining fertility rates observed in developed nations have raised concerns among policymakers, academics and the media due to their demographic, social, and economic implications. This trend has sparked a debate about the role of public policies for fertility, recently influenced by empirical evidence of a mismatch between desired and actual fertility [1]. Most governments of low-fertility countries have implemented social policies explicitly aimed at increasing fertility rates [2]. Such policies frequently focus on monetary incentives and longer, subsidized maternity leaves [3], favoring, as the arena for childbearing, a division of gender roles between parents, with men as main providers and women as main carers. Such arrangements are increasingly undesired or economically unsustainable for couples in contemporary developed societies. Critically, pronatalist policies overlook the importance of work-life balance for (prospective) parents in fertility decisions. Yet, recent empirical literature on family ideals indicates that younger cohorts prioritize the absence of work-family conflict and the avoidance of a double-burden for women [4], as well as higher life satisfaction is found to be associated with higher probability of having a first or second child [5].

The last two decades have witnessed an increasing effort by European Union institutions, as well as many Member States, to foster a ‘social investment’ agenda. Early childhood education and care (ECEC) services have been at the forefront of this European strategy, such as the Child Care Guarantee in the European Pillar of Social Rights [6]. While many EU countries have witnessed an increase in ECEC coverage and public expenditure, most countries still have not reached the targets. In 2002, through the “Barcelona target”, Member States agreed to increase the share of children under three in formal childcare to 33% by 2010, yet only twelve countries met this goal [7]. The target was revised in 2022 to 45% by 2030 and renamed European Strategy, but, at the time of writing, only 3 countries have met this threshold [8,9].

Expanding public childcare provision has been shown to increase mothers’ labor market re-entry after childbirth, favor parents’ work-family reconciliation [10], lower the cost of children [11,12], and foster equality among partners [13]. Hence, an increase in the provision of public childcare may contribute to removing some of the barriers which prevent couples from achieving their desired fertility aspirations [3], may influence fertility decision-making [14] and, ultimately, contribute to increasing fertility levels [15].

The predominant result from recent reviews is that formal childcare (i.e., targeted to children aged 0–2 years) availability and generosity have, in general, a positive and lasting effect on childbearing [15,16]. However, empirical findings are mixed, depending on the context and measures of childcare and fertility used [17]. While some studies show a positive, albeit moderate, association between childcare and fertility [18,19], even after accounting for selection into childcare usage [20], others find no association [21,22]. Furthermore, the association, when observed, tends to be stronger among higher-educated women [18,23,24], likely due to their greater opportunity costs of labor market withdrawal [25]. Conversely, some studies find no educational gradient in the association [19]. Moreover, previous literature suggests that increases in childcare are more strongly associated with fertility in contexts where usage is relatively low, given its importance in reducing work-family conflicts, especially for mothers, since these are contexts where the female labor market is still lagging behind, compared to contexts in which public childcare is the norm [11,26,27].

Existing literature on childcare and fertility has primarily relied on single-country studies, largely due to the lack of comparable international data on childcare. Previous comparative studies employed national-level measures of childcare, often captured at a single point in time [18,19,23]. However, national-level measures might hide the fact that childcare provision, and use, may vary considerably over time and within countries, as decisions regarding this policy measure are generally administered at the regional, provincial, or municipal level. As a result, prior literature has mainly focused on fertility differences across institutional settings defined by their childcare scores, thereby failing to capture the dynamic association between increases in childcare and fertility, net of institutional similarities.

To address these limitations, this contribution measures childcare provision at the sub-national level and over time, moving beyond national level averages and introducing a novel combination of subnational and longitudinal perspectives in studying the relationship between childcare and fertility over 60 regions across 11 European countries.

We study *if*, *for whom*, and *where* an expansion in early childcare might be positively associated with fertility. First, we analyze the relationship between a change over time in the regional provision of public childcare and the transition to first and second births. Second, we address the heterogeneity of the associations at the micro level, across different socioeconomic statuses, focusing on differences by educational level and employment status. Third, we address macro-level heterogeneity stratifying the associations between early childcare and fertility by the average level of childcare in the region of residence.

We leverage yearly subnational data on childcare usage across Europe and individual-level longitudinal data on parity progression, by merging four-year longitudinal individual-level data from the European Union Statistics on Income and Living Conditions (EU-SILC, 2005–2020) with annual data on unique public childcare usage [28]. This approach allows accounting for within-country heterogeneity while holding country-specific institutional factors constant. To the best of our knowledge, this is the first cross-country study on public childcare and fertility that analyzes variation below the national level. Our final sample consists of 10,593 childless women and 20,171 mothers of a sole child, resulting in two samples of 35,894 and 66,696 person-year observations, respectively. The list of included countries and the exclusion criteria can be found in Supporting Information, S1 File*,* Table S1.

First, we present results on the overall association between public early childcare expansion and first- or second-parity transitions. Second, we explore micro-level heterogeneity by introducing interactions with women’s level of education and with employment status. Finally, we assess macro-level heterogeneity by examining the interaction with the overall regional average of childcare usage, measured across the study period.

## Materials and methods

### Data

The analysis covers 60 sub-national regions, from 11 European countries for which we can observe information on public ECEC drawn from administrative data measured at the sub-national level and survey microdata on fertility measured at the individual-level, spanning from 2005 to 2020. The list of included countries and the exclusion criteria can be found in the Supporting Information*,* S1 File, Table S1. Micro-level data on childbirth and other individual-level characteristics are drawn from the European Union Statistics on Income and Living Conditions longitudinal data (EU-SILC), characterized by a 4-year rotating panel design [29,30]. Data were first accessed on 12^th^ of December 2022 for research purpose and authors had no access to information that could identify individual participants to the study.

Data on public early childcare usage are recorded yearly at the regional level, between 2000 and 2020 [28]. These data record administratively registered 0–2 years childcare usage in the region at different levels of aggregation, depending on the country. Public usage can be interpreted as a proxy for availability or coverage, given the general undersupply. It also indicates the level of generosity and universalism of early childcare policy implementation.

The EU-SILC level of aggregation (NUTS level) of the region of residence varies between countries. Since, in some countries, NUTS boundaries have changed between 2005 and 2020, we combined regions by computing population-weighted averages of early childcare usage and excluded countries lacking sub-national residence data in EU-SILC, which are listed in Supporting Information, S1 File, Table S1. Sensitivity analyses confirm that including these countries using national-level childcare measures does not alter our main findings.

In line with previous research, because being in a partnership is frequently a precondition for childbearing, the final sample conditions on the presence of a partner and includes women 20–45 years old [31,32]. Moreover, we excluded from our sample respondents in education, retired, or permanently disabled.

Our final sample consists of 10,593 childless women and 20,171 mothers of one child, resulting in two samples of 35,894 and 66,696 person-year observations, respectively.

### Measures

Taking advantage of EU-SILC’s panel design, we define first- and second-birth transitions as time-to-event binary outcomes observed over a 4-year panel period. Our approach relies on the own-children method, identifying women’s parity based on the number of children living in their household at the time of the interview. For the transition to parenthood, we include only childless women at the start of the observation period (t=0). For each subsequent year (t=1, 2, 3), we recode transition to parenthood to equal 1 if a woman transitions from zero to one co-residing child, indicating her first birth; otherwise, the first-parity outcome remains 0. Similarly, the transition to a second birth is measured on women with exactly one co-residing child at the start of the observation period (t=0). Then, for each subsequent year, second-parity transitions are recoded to 1 if a woman transitions from one to two co-residing children, indicating her second birth; otherwise, the second-parity outcome is 0.

The main explanatory variable is the yearly regional rate of public early childcare (ECEC) usage for children aged 0–2, demeaned at the regional level and lagged by two years. This is defined as the number of children aged 0–2 enrolled in publicly subsidized childcare divided by the total number of children aged 0–2 in the region, and captures actual enrolment rather than the number of available slots. Actual enrolment, however, also serves as a proxy for availability given the general undersupply of public early childcare documented across European regions.

### Statistical methods

Given the sub-national level of geographical aggregation of our data, we apply regional-level demeaning to isolate the association with variation in childcare usage within the region of residence, while accounting for fertility differences driven by overall childcare levels – i.e., we measure ECEC usage in the region of residence as a deviation from its overall regional average, computed over the study period [33]. This approach addresses between-region heterogeneity while accounting for institutional similarities. To reduce the likelihood of reverse causality, ECEC is lagged by two years, although this does not, in principle, preclude anticipation effects, whereby couples may adjust fertility intentions in response to announced childcare expansions in later years. Furthermore, due to the time-to-event nature of our outcomes, we estimate associations using generalized linear models with a complementary log-log link, heteroskedasticity-robust standard errors clustered on individuals, accounting for longitudinal survey weights [34].

We developed four models to assess *if* (M1), *for whom* (i.e., for which educational level, M2, and employment status, M3), and *where* (i.e., in which regions, M4)*,* early childcare usage is associated with women’s transitions to first and second birth, defined as follows:


$$\begin{array}{c} Y_{ijk}=\beta_{\text{0}}+\overset{dm}{\underset{jk}{\beta_{\text{1}}ECEC\;}}+{\gamma X}_{ijk}+\epsilon_{ijk} \end{array}$$ (1)

$$\begin{array}{c} Y_{ijk}=\beta_{\text{0}}+\beta_{\text{1}}ECECjkdm+\beta_{\text{2}}Eduijk\;+\beta_{\text{3}}ECECjkdm\times Eduijk\;+\gamma Xijk\;+\epsilon_{ijk} \end{array}$$ (2)

$$\begin{array}{c} Y_{ijk}=\beta_{\text{0}}+\beta_{\text{1}}ECECjkdm+\beta_{\text{2}}Emplijk\;+\beta_{\text{3}}ECECjkdm\times Emplijk\;+\gamma Xijk\;+\epsilon_{ijk} \end{array}$$ (3)

$$\begin{array}{c} Y_{ijk}=\overset{dm}{\underset{jk}{\beta_{\text{0}}+\beta_{\text{1}}ECEC}}+\overset{m\;}{\underset{k}{\beta_{\text{2}}ECEC}}+\overset{m^{\text{2}}}{\underset{k}{\beta_{\text{3}}ECEC}}+\overset{dm}{\underset{jk}{\beta_{\text{4}}ECEC}}\times \overset{m}{\underset{k}{ECEC}}+\overset{dm}{\underset{jk}{\beta_{\text{5}}ECEC}}\times \overset{m^{\text{2}}}{\underset{k}{ECEC}}+{\gamma X}_{ijk}+\epsilon_{ijk} \end{array}$$ (4)

where $\overset{dm}{\underset{jk}{ECEC}}={ECEC}_{jk}-{E\left[{ECEC}_{j}\right]}_{k}$ is the level of childcare usage ($E{CEC}_{jk}$) for every year *j* and region *k,* lagged by two years, and demeaned at the regional level by ${EC\overset{m\;}{\underset{k}{EC}}=E\left[{ECEC}_{j}\right]}_{k}$. $X_{ijk}$is the matrix of controls and γ the associated vector of coefficients. All models are adjusted for age (five-year categories), civil status (marriage dummy), region of residence (dummies), year of entry in the study (dummies), education level (lower secondary, upper secondary, and tertiary), and employment status (not employed and employed), with the exception of M2, where we do not control for employment status, and M4, where we control for country dummies instead of region of residence to avoid perfect collinearity with $\overset{m}{\underset{k}{ECEC}}$. The distribution of the covariates in our samples can be found in Supporting Information, S1 File, Table S2. Region-of-residence dummies absorb time-stable differences across regions in fertility behavior, labor market structure, and family policy context, while year-of-entry dummies account for common temporal trends affecting all regions simultaneously. Together, they reduce concerns about confounding by stable compositional differences and aggregate period shocks. However, this strategy cannot account for region-specific time-varying unobserved factors, such as concurrent regional changes in labor market conditions or family support policies other than ECEC expansion.

Results are also presented as the increase in hazards associated with a 10 units increase in ECEC. Because hazard ratios are derived from exponentiating the model coefficient ($HR=e^{\beta }$), the hazard ratio for a 10 units increase is obtained by exponentiating the coefficient after multiplying it by 10 (equivalently, the 1-point hazard ratio is raised to the 10th power): $HR_{\text{1}\text{0}}=e^{\text{1}\text{0}\beta }=HR^{\text{1}\text{0}}$.

## Results

### Descriptive results

**Fig 1:**
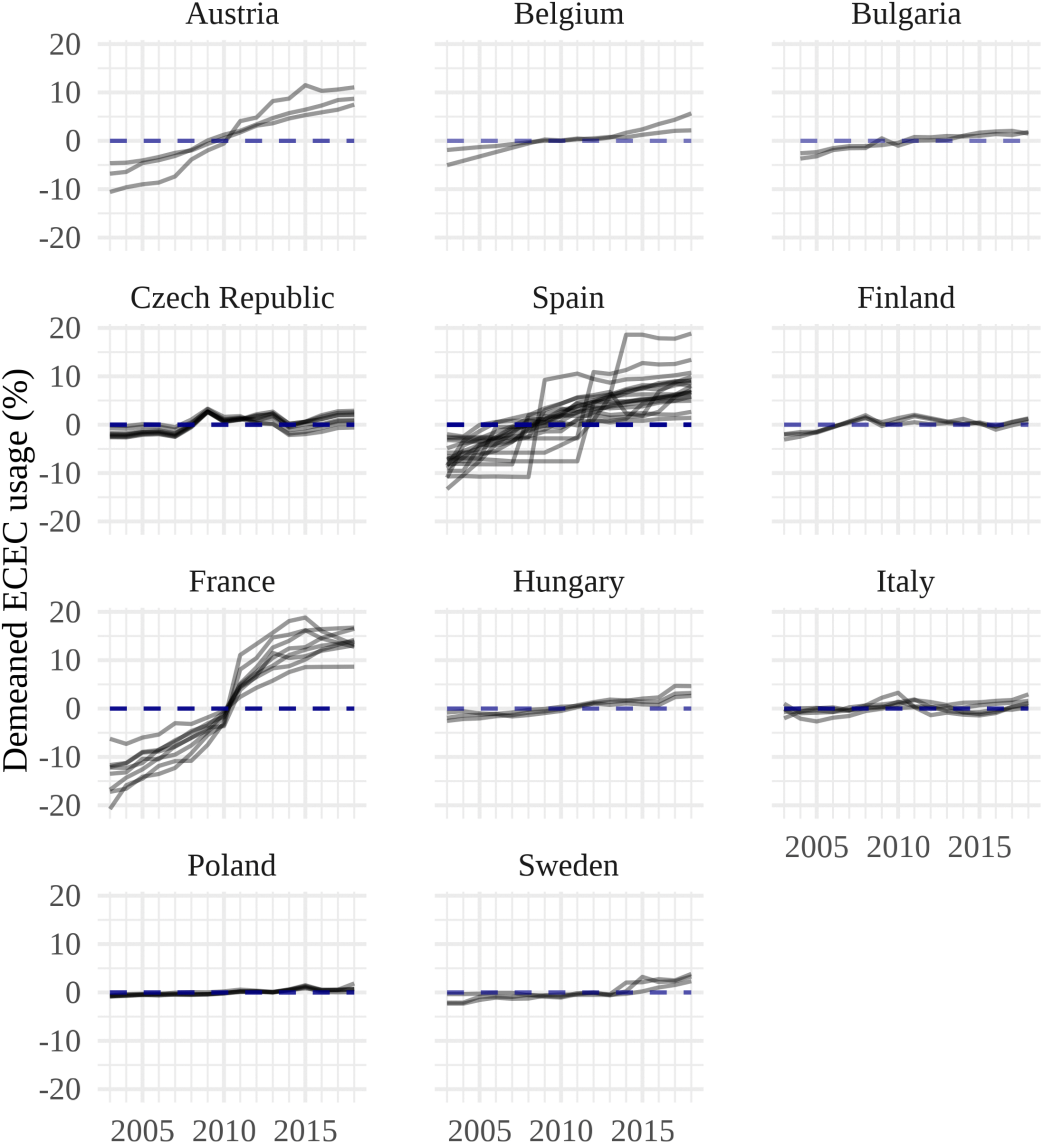
Yearly regional trends of demeaned average childcare usage, grouped by country, from 2003 to 2018. Demeaned childcare usage can also be interpreted as variations from the overall regional mean. The dashed line indicates levels at which the regional average of childcare usage is equal to the overall mean across the observational window. We present yearly regional trends from 2003 to 2018 since childcare usage enters our models lagged by two years (t-2) with respect to the indicator of parity transition.

Public childcare in Europe has increased, but at different rates. Public childcare not only varies considerably across European regions in its level, ranging from 0% in 2003 in five regions of Spain to 84.7% in the Ouest region of France (for detailed regional information on childcare usage regional trends see Scherer & Pavolini, 2023) [28], but also in its trends over the last 15 years (Supporting Information, S1 Fig). Fig 1 presents regional trends of average childcare, demeaned at the regional level, from 2003 to 2018. By subtracting from the yearly measures, the overall regional mean of the study period, we isolate within-regions variations in ECEC usage, accounting for differences in levels across regions. While ECEC usage increased in most observed regions, the extent of this increase varied substantially. Only some regions of Austria, France and Spain have experienced an increase in childcare usage that is greater than 20%, while most regions have experienced between 5 and 10% increases, from 2003 to 2018. In contrast, most regions in the Czech Republic, Finland, Italy, and Poland have shown little to no variation across the study period, with regional childcare fluctuating around the overall mean for each region. In the Czech Republic and Finland, the slow expansion of ECEC for children aged 0–2 may somewhat reflect the introduction of long parental leave schemes and cash-for-care benefits, which might keep mothers out of the labor market when public childcare is scarce or expensive [35], thus potentially reducing formal care use among infants [36,37].

### Multivariate analysis

**Fig 2:**
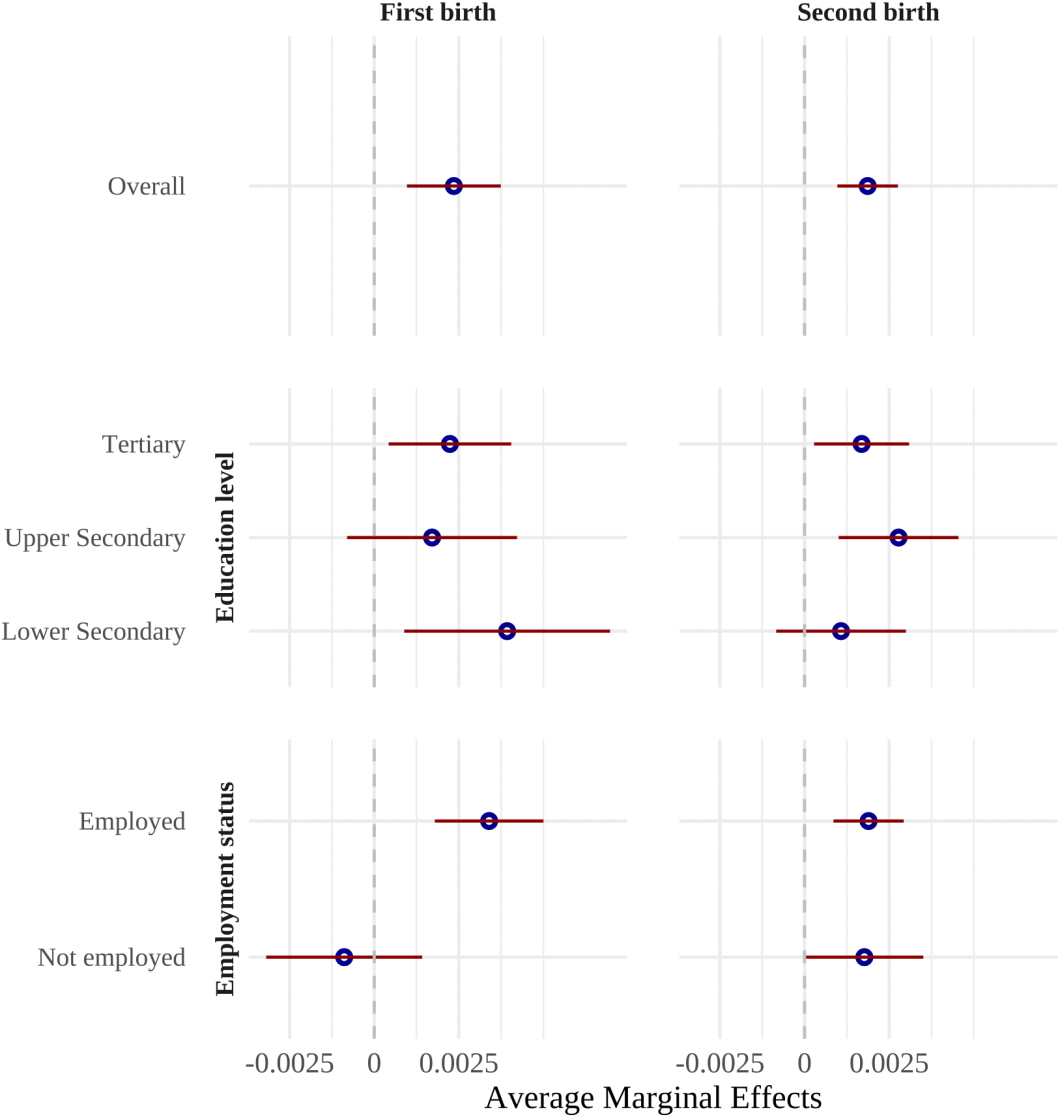
Associations between demeaned lagged childcare usage and first (*left*) and second (*right*) parity transitions overall (*top*), by education level (*middle*), and by employment status (*bottom*). Point estimates are Average Marginal Effects of demeaned childcare resulting from six *cloglog* regressions adjusted for age, marital status, region of residence, year of entry in the survey, level of education, and employment status. Error bars are 95% CIs based on heteroskedasticity-robust SEs clustered at the individual level. Positive values indicate positive associations, while negative associations are denoted by negative values. These associations are said to be statistically significant when error bars do not overlap the dashed (zero) line.

**Fig 3:**
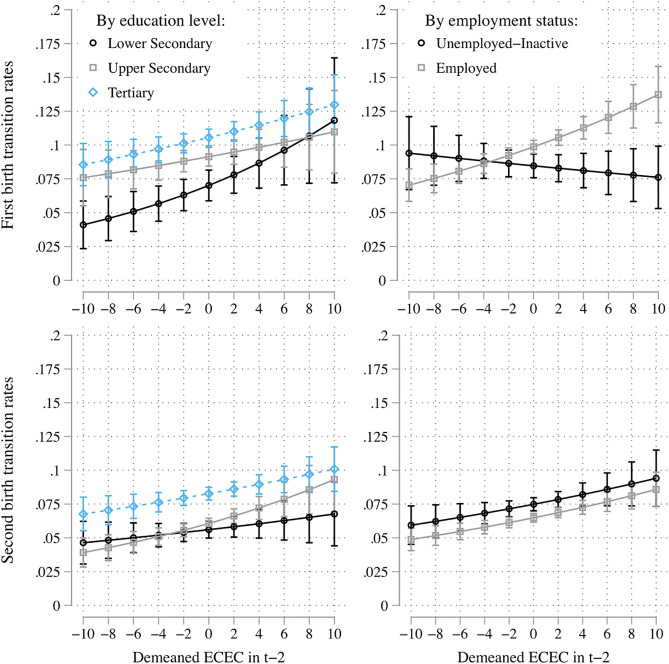
Estimated adjusted rates, i.e., conditional probabilities, of first (*top*), and second (*bottom*), parity transitions by demeaned lagged childcare usage, by education level (*left*) and employment status (*right*). Point estimates are the rates resulting from four *cloglog* regressions adjusted for age, marital status, region of residence, year of entry in the survey, level of education, and employment status. Error bars are 95% CIs based on heteroskedasticity-robust SEs clustered at the individual level. The rate measures the number of events divided by the number of person-years at risk. It can be interpreted as a conditional probability of experiencing the event, conditioned on not having experienced the event yet.

**Fig 4:**
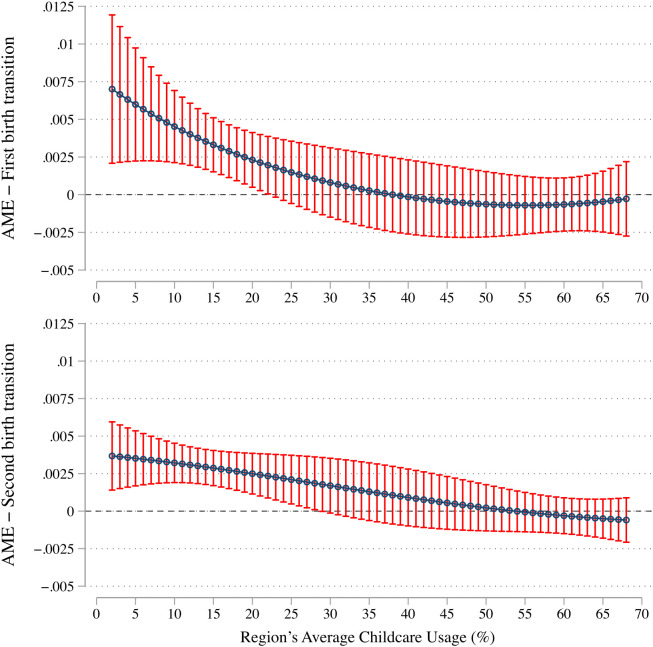
Associations between demeaned lagged childcare usage and first (*left*) and second (*right*) parity transitions overall (*top*), by overall regional average ECEC usage over the study period. Point estimates are Average Marginal Effects of the demeaned childcare interacted with regional overall ECEC usage squared, resulting from two *cloglog* regressions adjusted for age, marital status, region of residence, year of entry in the survey, level of education, and employment status. Error bars are 95% CIs based on heteroskedasticity-robust SEs clustered at the individual level. Positive values indicate positive associations, while negative associations are denoted by negative values. These associations are said to be statistically significant when error bars do not overlap the dashed (zero) line.

Details on the association between these ECEC changes within regions and fertility, distinguishing transitions to first and second births, are reported in Figs 2–4. Fig 2 assesses whether first and second birth rates do respond to changes in ECEC, presenting the direction and magnitude of the associations in terms of average marginal *effects*. Fig 3 displays predicted *rates* of births as ECEC increases. Both figures report results overall and stratified by level of education and by employment status. Variation over time in regional childcare is measured two years prior to the fertility measure. Fig 4 reports the association between an ECEC increase and first- and second-birth transitions at different average levels of regional ECEC, reported as average marginal *effects.* Results are derived from eight adjusted complementary log-log regressions (see the *Materials and Methods* section; models’ coefficients are provided in Supporting Information, S1 File, Tables S4 and S5).

Public childcare expansions are associated with higher fertility. The top panels of Fig 2 quantify the increase in first- and second-birth transition rates as public childcare in the region of residence increases, net of relevant controls. One percentage point increase in ECEC is associated with an increase in first-birth rates of 0.24 points (AME=0.0024 ,  P=0.001) and in second-birth rates of 0.19 points (AME=0.0019,  P<0.001). Results show a clear positive association between a previous increase in public ECEC and first and second births, though the rates of transitioning to a first child are higher than those of a second child (Fig 3). First- and second-birth transitions occur at low rates in our sample with unadjusted first- and second-birth rates equal to 0.098 and 0.071, respectively (Supporting Information, S1 File, Table S3). Because baseline rates are small, modest absolute changes translate into substantial percentage increases. Increasing ECEC by 10 percentage points is associated with an increase of 29% and 32% in the transition rates to first and second births respectively (HR=1.026 ,  P=0.001; HR=1.028 ,  P<0.001). This translates to an increase in transition rates from 9.8 to 12.2 first births per 100 women and from 7.1 to 9.0 second births.

The lower panels of Fig 2 assess to what extent these associations vary by women’s level of education and employment status. Fertility transitions follow a clear educational gradient with tertiary-educated women experiencing higher first- and second-birth transition rates than lower-secondary-educated women, and higher second-birth transition rates than upper-secondary-educated women (Fig 3). Thus, our findings align with recent literature highlighting the emergence of social inequalities in fertility, with lower-educated women being less likely to enter motherhood than higher-educated women [38–40]. As ECEC increases, fertility is higher, but lower-secondary-educated women experience a steeper increase in first-birth transition rates, while upper-secondary-educated women show a steeper increase in second-birth transition rates, levelling off partially the disparities in fertility levels across women with different levels of education. The association between an increase in ECEC usage and becoming a first-time mother is significant only among tertiary-educated (AME=0.0022,  P=0.015) and lower-secondary-educated women (AME=0.0039,  P=0.011), who exhibit the largest increase (Fig 2). An increase of 10 percentage points from the overall regional ECEC level is associated with almost a 75% increase in the rates of becoming mothers among lower-secondary-educated women (HR=1.057, P=0.006). We find no associations with second-birth transitions among lower-secondary-educated women (Fig 2), while upper-secondary-educated mothers display the largest estimate (AME=0.0028,  P=0. 002), among whom an increase of 10 percentage points in ECEC is associated with a 57% increase in the rates of second-parity transitions (HR=1.046,  P=0.001).

First-birth rates diverge between employed and non-employed women as regional childcare usage increases, whereas second-birth rates show minimal differences by employment status (Fig 3). The bottom panels of Fig 2 report differences in the association by employment status: an increase in ECEC is positively associated with first-birth transitions only among employed women (AME=0.0034,  P<0. 001), while no significant association is observed among non-employed women (AME = −0.0009, P = 0.450). The absence of an association among non-employed women for first-birth transitions suggests that, where labor market attachment is weak, ECEC expansions do not appear to lower the barriers to parenthood. ECEC expansions however are positively associated with second-birth transition rates among both employed and non-employed mothers (AME=0.0019,  P<0. 001, and AME=0.0018,  P=0.047, respectively). An increase of 10 percentage points in public ECEC is associated with a 43% increase in first-birth rates among employed women and a 34% increase in second-birth transitions among employed mothers (HR=1.037,  P<0.001 and HR=1.030,  P<0.001, respectively), reducing to 28% among non-employed mothers (HR=1.024,  P=0.042).

The distribution of ECEC across regions is quite uneven, with 25% of regions in our sample reporting an overall average ECEC below 6%, while 50% are below 12%, and 75% are below 23% (Supporting Information, Fig S2). Fig 4 shows that the association between increased ECEC and birth transitions depends on the overall level of ECEC usage. In regions where public ECEC exceeds 22% overall (i.e., 75% of the regions in our sample), we find no statistically significant association between childcare expansions and first-birth transitions (ECEC =23%: AME=0.0018,  P=0.073). By contrast, in regions where 22% or less of eligible children are enrolled in public ECEC the association is positive and statistically significant (ECEC usage =22%: AME=0.0020,  P=0.044). The same pattern is observed for second births, yet the association is statistically significant in regions where public ECEC enrolment is up to 27% overall (ECEC usage =27%:  AME=0.0019, p=0.038).

## Discussion

This study provides evidence that an increase in publicly subsidized early childcare and education services for children below the age of 3 is associated with higher fertility, and it does so mainly in contexts characterized by overall low levels of childcare provision.

Fertility is measured by parity-specific transitions to first and second births while public childcare provision is proxied by the share of children enrolled in early childcare relative to the total number of eligible children, measured at the regional level on a yearly basis. This allows accounting for the substantial within-country variation in both childcare usage and fertility behaviors, which was hidden in national-level averages and fertility trends.

Publicly subsidized childcare supports mothers’ continued employment or eases labor market re-entry [14], which improves household income, and promotes work-family balance [10,12]. These factors are likely pathways underlying the reported positive associations, which have been found to promote total fertility and reduce postponement [41–43]. In addition, an increase in public childcare also indicates state investment in families and work-family reconciliation, providing concrete signals.

The positive association between public childcare and childbearing, however, does not hold for everyone, nor everywhere to the same extent, but we document considerable heterogeneity between contexts and, to a lesser extent, between social groups.

Public childcare is positively associated with both parenthood and second-birth transitions among women with a university degree, whereas among lower-secondary-educated women the association is observed only for the transition to parenthood and among upper-secondary-educated women only for the transition to a second birth. While tertiary-educated women, theoretically, should be more sensitive to reductions in opportunity costs by service expansion due to their overall higher opportunity cost of motherhood [44], they also have greater economic resources to afford private solutions, if needed. Less-educated women, instead, face more financial constraints, thus being more reactive to affordable public childcare expansions, which, by fostering maternal employment [45], contribute to increasing household income, thereby improving couples’ ability to face economic shocks such as the cost of a child [46,47]. Empirically, we see the outcome of both counteracting processes. Women’s employment situation moderates the childcare effects, with only employed women showing higher rates of becoming a parent as childcare services grow, but a positive association with second-order parities is observed for both employed and non-employed mothers. If public childcare is available only for women who are employed, or if fees apply that make it less convenient than mothers’ foregone earnings, then inactive and unemployed women, especially in lower socioeconomic strata, will forgo public childcare, despite its availability.

Expanding ECEC is positively associated with fertility in regions where public childcare is scarce, while its further increase does not come with effects in regions where ECEC is already common, indicating a “saturation-effect”. We find this threshold in the regional average of childcare usage to be 22%, in analyzing the relationship with first-birth transitions, which becomes 27% in analyzing the relationship with second-birth transitions. Regional average childcare is above this 22% threshold only in one out of four regions in our sample, suggesting that expanding public, hence affordable, childcare could increase fertility in many regions. These thresholds however reflect the distributional structure of our analytical sample and should be interpreted as indicative benchmarks rather than universal policy standards, given that existing policy targets are informed by broader criteria beyond the childcare-fertility association alone.

Just like any single family policy, expanding public childcare alone is not going to increase fertility rates dramatically, but at the same time these services seem to make a difference: according to our findings, an increase of 10% in the share of children in public childcare is associated with an increase in first-birth transition rates from 9.8 to 12.2 births per 100 women, and from 7.1 to 9.0 second births, in the following four years – equivalent to about a one-third increase in transition rates. What is more, childcare expansions might help in reducing the mismatch between desired and actual fertility, as indicated by higher short-term parity transition rates. Whether these associations persist and translate into higher completed fertility calls for future research employing longer follow-up windows. Coupled with childcare’s positive contribution to increasing mothers’ participation and gender equality in the labor market [28,45,48,49], this makes the Council of the European Union’s recommendation that Member States reach a target of 45% of children under three in childcare by 2030 particularly timely [50].

This study presents a set of limitations and we performed, when possible, several robustness checks to address the validity of our results (available upon request). Thus, we do not intend to make any causal claim, but rather to fill the gap in previous literature on the association between public childcare and fertility. In particular, while lagging ECEC by two years reduces reverse causality concerns, anticipation effects cannot be ruled out: couples may adjust fertility plans in response to announced expansions before they materialize.

First, given that fertility is measured using the own children method (see the *Materials and methods* section), children living outside the household could introduce misclassification bias. To mitigate this, we restricted the sample to women under 45 and tested whether our results are robust to changes in the sample’s age threshold, restricting maximum age to 35, 40, and 49 years, finding no significant difference in the estimated coefficients. Although misclassification bias in the own-children method is unlikely to bias fertility estimates substantially [32,51], residual misclassification might be unevenly distributed across socioeconomic groups [31]. Second, the short panel framework provided by EU-SILC comes with drawbacks stemming from left and right censoring, limiting our analysis to short-term parity transitions. However, it has been successfully employed by previous literature studying fertility behaviors [18,52,53]. Third, results might be biased by single countries driving the overall associations. To test for this and to support the choice of demeaning our macro-level explanatory variable, we conducted a leave-one-country-out analysis and estimated country-specific results. We confirmed the robustness of our results and found little heterogeneity between estimates. Fourth, our results might suffer from selection bias due to the exclusion from our analysis of countries lacking regional information at the subnational level (Supporting Information, S1 File, Table S1). We have tested this by running our results including those countries based on national-level childcare measures, yielding similar results. Fifth, we measure childcare usage rather than availability. An expansion in childcare *usage*, however, captures families’ effective access to public childcare. This might signal interventions that improve access to childcare services or reduce their cost, such as income-based progressive fees, even if the number of available childcare slots is constant. Moreover, childcare usage might reflect not only the supply-side capacity and eligibility rules, but also a combination of prevailing gender norms around infant childcare enrolment, labor market changes, and parental preferences, meaning that the estimated associations may partly capture broader institutional and societal changes rather than childcare availability alone. Additionally, although region and year dummies absorb time-stable regional heterogeneity and a common time trend, they cannot account for region-specific time-varying unobserved confounders, such as regional shifts in labor market conditions, parental leave provision, or other family policies. Lastly, since birth transitions are rare events in our sample, we tested different regression families: logistic, negative binomial, and Poisson regressions with heteroskedasticity-robust standard errors clustered on individuals. All models showed similar estimates to those produced using GLMs with a complementary log-log link, which are to be preferred when outcomes are time-to-event variables and reduce rare event bias [34].

## Conclusion

Social policies with the explicit aim to raise fertility were implemented in most low-fertility countries, usually involving monetary incentives and subsidized maternity leaves, but these policies frequently overlook the importance of work-life balance for (prospective) parents. Increasing the provision of early child education and care services instead not only invest explicitly in the future generation but is also associated with greater gender equality, which in turn has been linked with higher fertility. Overall, our results provide a clear picture of the role of publicly subsidized childcare in supporting transitions to parenthood and to second births, especially in regions where said services are insufficient (i.e., three quarters of the regions in our data, that show an average childcare usage below 23% and have not departed from such threshold by 2019), and thus where public investment is most needed. Thus, investing in childcare services, a family policy that promotes dual-earner couple arrangements with a certain degree of work-family balance, could make a difference for the fertility decline observed in lowest-low fertility contexts and among less advantaged women, who are found to be less involved in the labor market [13] and characterized by lower fertility and high levels of childlessness [39]. Most importantly though, public childcare appears to be an instrument enabling persons to have the number of children they desire, while not having to give up employment.

## Supporting information

S1 Fig Yearly regional public childcare ECEC usage by country. The bold line is the yearly national average. Authors elaboration on data from Scherer S, Pavolini E. Equalizing or not? Public childcare and women’s labor market participation. Journal of European Social Policy. 2023. https://doi.org/10.1177/09589287231183169.
(TIF)

S2 Fig Boxplot of regional overall public childcare usage. The boxplot displays the median, the interquartile range, and whiskers extending to the most extreme values within 1.5 times the interquartile range, with regions beyond this threshold shown as individual outliers. Authors elaboration on data from Scherer S, Pavolini E. Equalizing or not? Public childcare and women’s labor market participation. Journal of European Social Policy. 2023. https://doi.org/10.1177/09589287231183169.
(TIF)

S1 File Description of the sample and regression coefficients. S1–S5 Tables.
(PDF)

## References

[1] Beaujouan É, Reimondos A, Gray E, Evans A, Sobotka T. Declining realisation of reproductive intentions with age. Hum Reprod. 2019;34(10):1906–14. doi: 10.1093/humrep/dez150 31560763

[2] Gietel-Basten S, Rotkirch A, Sobotka T. Changing the perspective on low birth rates: why simplistic solutions won’t work. BMJ. 2022. doi: 10.1136/bmj-2022-072670 PMC10915774 36379520

[3] UNFPA. Annual Report 2022-23. New York, USA: United Nations Population Fund. 2023. https://www.unfpa.org/sites/default/files/pub-pdf/UNFPA-2022-23AR-EN-Final.pdf

[4] Aassve A, Adserà A, Chang PY, Mencarini L, Park H, Peng C, et al. Family ideals in an era of low fertility. Proc Natl Acad Sci U S A. 2024;121(6):e2311847121. doi: 10.1073/pnas.2311847121 38294942 PMC10861923

[5] Mencarini L, Vignoli D, Zeydanli T, Kim J. Life satisfaction favors reproduction. The universal positive effect of life satisfaction on childbearing in contemporary low fertility countries. PLoS One. 2018;13(12):e0206202. doi: 10.1371/journal.pone.0206202 30517115 PMC6281189

[6] European Commission, European Education and Culture Executive Agency. Key data on early childhood education and care in Europe 2025 – Eurydice report. Publications Office of the European Union. 2025. doi: 10.2797/66224

[7] European Commission JD-G for, Hoorens S, Rendall M, Tsang F. Gender equality in the workforce – Reconciling work, private and family life in Europe. Publications Office. 2014. doi: doi/10.2838/54302

[8] European Commission. A European Care Strategy. European Commission - European Commission. https://ec.europa.eu/commission/presscorner/detail/en/ip_22_5169 2022. Accessed 2025 August 12.

[9] Eurostat. Formal child care by duration and age group. Eurostat. 2025. doi: 10.2908/TPS00185

[10] Thévenon O, Gauthier AH. Family policies in developed countries: a ‘fertility-booster’ with side-effects. Community, Work & Family. 2011;14(2):197–216. doi: 10.1080/13668803.2011.571400

[11] Doepke M, Hannusch A, Kindermann F, Tertilt M. The economics of fertility: A new era. National Bureau of Economic Research. 2022. doi: 10.3386/w29948

[12] Thévenon O. Family policies in OECD countries: a comparative analysis. Popul Dev Rev. 2011;37(1):57–87. doi: 10.1111/j.1728-4457.2011.00390.x 21714199

[13] Hook JL, Li M. Gendered tradeoffs. In: Nieuwenhuis R, Van Lancker W, editors. The Palgrave Handbook of Family Policy. Cham: Springer International Publishing. 2020:249–66. doi: 10.1007/978-3-030-54618-2_11

[14] Billingsley S, Neyer G, Wesolowski K. Social investment policies and childbearing across 20 countries: Longitudinal and micro-level analyses. European Journal of Population. 2022;38:951–74. doi: 10.1007/s10680-022-09626-3 36507245 PMC9727052

[15] Bergsvik J, Fauske A, Hart RK. Can Policies Stall the Fertility Fall? A Systematic Review of the (Quasi‐) Experimental Literature. Population & Development Rev. 2021;47(4):913–64. doi: 10.1111/padr.12431

[16] Luci-Greulich A, Thévenon O. The Impact of Family Policies on Fertility Trends in Developed Countries: L’influence des politiques familiales sur les tendances de la fécondité des pays développés. Eur J Population. 2013;29:387–416. doi: 10.1007/s10680-013-9295-4

[17] Scherer S, Pavolini E, Brini E. Formal childcare services and fertility: the case of Italy. Genus. 2023;79:29. doi: 10.1186/s41118-023-00208-7

[18] Baizan P, Arpino B, Delclòs CE. The Effect of Gender Policies on Fertility: The Moderating Role of Education and Normative Context. Eur J Popul. 2016;32(1):1–30. doi: 10.1007/s10680-015-9356-y 27069290 PMC4803818

[19] Wood J, Neels K, Vergauwen J. Economic and Institutional Context and Second Births in Seven European Countries. Popul Res Policy Rev. 2016;35:305–25. doi: 10.1007/s11113-016-9389-x

[20] Wood J. Does formal childcare uptake stimulate fertility? Formal childcare usage and second births. Popul Res Policy Rev. 2025;44:42. doi: 10.1007/s11113-025-09963-1

[21] Del Boca D, Pasqua S, Pronzato C. Motherhood and market work decisions in institutional context: a European perspective. Oxford Economic Papers. 2009;61:i147–71. doi: 10.1093/oep/gpn046

[22] Schuss E, Azaouagh M. The expansion of early childcare and transitions to first and second birth in Germany. Bulletin of Econ Res. 2023;75:476–507. doi: 10.1111/boer.12367

[23] Van Bavel J, Rozanska Putek J. Second birth rates across Europe: interactions between women’s level of education and child care enrolment. Populationyearbook. 2010;8:107–38. doi: 10.1553/populationyearbook2010s107

[24] Wood J. Social differentials in the effect of formal childcare on the transition to parenthood?. Advances in Life Course Research. 2019;42:100309. doi: 10.1016/j.alcr.2019.100309 36732972

[25] England P, Bearak J, Budig MJ, Hodges MJ. Do highly paid, highly skilled women experience the largest motherhood penalty? Am Sociol Rev. 2016;81:1161–89. doi: 10.1177/0003122416673598

[26] Hilgeman C, Butts CT. Women’s employment and fertility: a welfare regime paradox. Soc Sci Res. 2009;38(1):103–17. doi: 10.1016/j.ssresearch.2008.08.005 19569295

[27] Korpi W, Ferrarini T, Englund S. Women’s opportunities under different family policy constellations: gender, class, and inequality tradeoffs in western countries re-examined. Social Politics: International Studies in Gender, State & Society. 2013;20:1–40. doi: 10.1093/sp/jxs028

[28] Scherer S, Pavolini E. Equalizing or not? Public childcare and women’s labour market participation. Journal of European Social Policy. 2023. doi: 10.1177/09589287231183169

[29] Borst M, Wirth H. EU-SILC Tools: eusilcpanel_2020 - first computational steps towards a cumulative sample based on the EU-SILC longitudinal datasets; Update. GESIS Papers. 2022. doi: 10.21241/SSOAR.79965

[30] Eurostat. Eurostat regional yearbook: 2022 edition. 2022 edition ed. Luxembourg: Publications Office of the European Union. 2022.

[31] Greulich A, Dasré A. Observing the number of children with EU-SILC: A quantification of biases. Population. 2019;73:685–720. doi: 10.3917/popu.1804.0719

[32] Krapf S, Kreyenfeld MR. Fertility assessment with the Own-Children Method: a validation with data from the German Mikrozensus. Rostock: Max Planck Institute for Demographic Research. 2015. doi: 10.4054/MPIDR-TR-2015-003

[33] Giesselmann M, Schmidt-Catran AW. Getting the within estimator of cross-level interactions in multilevel models with pooled cross-sections: Why country dummies (sometimes) do not do the job. Sociological Methodology. 2019;49:190–219. doi: 10.1177/0081175018809150

[34] Box-Steffensmeier JM, Jones BS. Event history modeling: A guide for social scientists. Cambridge University Press. 2004.

[35] Olivetti C, Petrongolo B. The Economic Consequences of Family Policies: Lessons from a Century of Legislation in High-Income Countries. Journal of Economic Perspectives. 2017;31:205–30. doi: 10.1257/jep.31.1.205 28443651

[36] Bičáková A, Kalíšková K. (Un)intended effects of parental leave policies: Evidence from the Czech Republic. Labour Economics. 2019;61:101747. doi: 10.1016/j.labeco.2019.07.003

[37] Österbacka E, Räsänen T. Back to work or stay at home? Family policies and maternal employment in Finland. J Popul Econ. 2022;35:1071–101. doi: 10.1007/s00148-021-00843-4

[38] Jalovaara M, Neyer G, Andersson G, Dahlberg J, Dommermuth L, Fallesen P, et al. Education, Gender, and Cohort Fertility in the Nordic Countries. Eur J Population. 2019;35: 563–86. doi: 10.1007/s10680-018-9492-2 PMC6639448 31372105

[39] Lappegård T. Future fertility trends are shaped at the intersection of gender and social stratification. 2020.

[40] Mills M, Blossfeld HP. The Second Demographic Transition Meets Globalization: A Comprehensive Theory to Understand Changes in Family Formation in an Era of Rising Uncertainty. In: Evans A, Baxter J, editors. Negotiating the Life Course. Dordrecht: Springer Netherlands. 2013: 9–33. doi: 10.1007/978-90-481-8912-0_2

[41] Alderotti G, Vignoli D, Baccini M, Matysiak A. Employment Instability and Fertility in Europe: A Meta-Analysis. Demography. 2021;58(3):871–900. doi: 10.1215/00703370-9164737 33899914

[42] Balbo N, Billari FC, Mills M. Fertility in Advanced Societies: A Review of Research: La fécondité dans les sociétés avancées: un examen des recherches. Eur J Popul. 2013;29(1):1–38. doi: 10.1007/s10680-012-9277-y 23440941 PMC3576563

[43] Scherer S, Brini E. Employment instability and childbirth over the last 20 years in Italy. Eur J Population. 2023;39:31. doi: 10.1007/s10680-023-09680-5 PMC10570255 37823967

[44] Gauthier AH, Hatzius J. Family benefits and fertility: An econometric analysis. Population Studies. 1997;51:295–306. doi: 10.1080/0032472031000150066

[45] Hook JL, Paek E. National Family Policies and Mothers’ Employment: How Earnings Inequality Shapes Policy Effects Across and Within Countries. Am Sociol Rev. 2020;85(3):381–416. doi: 10.1177/0003122420922505 33612841 PMC7891546

[46] Barbieri P, Bozzon R. Welfare, labour market deregulation and households’ poverty risks: An analysis of the risk of entering poverty at childbirth in different European welfare clusters. Journal of European Social Policy. 2016;26(2):99–123. doi: 10.1177/0958928716633044

[47] Matysiak A, Vignoli D. Fertility and women’s employment: a meta-analysis. Eur J Population. 2008;24:363–84. doi: 10.1007/s10680-007-9146-2

[48] Pettit B, Hook JL. Gendered tradeoffs: family, social policy, and economic inequality in twenty-one countries. New York, N.Y.: Russell Sage Foundation. 2009.

[49] Kalíšková K, Münich D. The impact of childcare availability on maternal employment: Evidence from Czech municipalities. PLoS One. 2023;18(7):e0288987. doi: 10.1371/journal.pone.0288987 37471426 PMC10358966

[50] Council of the European Union. Early childhood education and care: the Barcelona targets for 2030. 2022. https://www.eumonitor.eu/9353000/1/j4nvk6yhcbpeywk_j9vvik7m1c3gyxp/vlz27fb07kyx

[51] Greulich A, Dasré A. The quality of periodic fertility measures in EU-SILC. Demographic Research. 2017;36:525–56. doi: 10.4054/DemRes.2017.36.17

[52] Hsu CH. How women’s employment instability affects birth transitions: the moderating role of family policies in 27 European countries. European Sociological Review. 2023. doi: 10.1093/esr/jcad037

[53] Nitsche N, Matysiak A, Van Bavel J, Vignoli D. Partners’ Educational Pairings and Fertility Across Europe. Demography. 2018;55(4):1195–232. doi: 10.1007/s13524-018-0681-8 29881980

