# Supplementary material for: Early public childcare and fertility: A longitudinal study for Europe
Source: PLoS One. 2026 Jul 14;21(7):e0353502. doi: 10.1371/journal.pone.0353502 (PMC13367713; doi:10.1371/journal.pone.0353502)
Supplement: S1 File — S1–S5 Tables. (PDF) [file pone.0353502.s003.pdf]

Supporting Information

Descriptives

Sample's descriptives

Childless women are more prevalent in lower age cohorts, with respect to mothers (S1 Table). They concentrate between 25 and 34 years old and only 22 percent of them are older than 40. Mothers are concentrated in the age group 30-39 and distributed toward older age groups (40-45) with respect to childless women. When entering the survey, childless women are evenly distributed between married and non-married couples, while married mothers are three times more prevalent than non-married ones. Moreover, most of the woman in both samples are employed – either part-time or full-time – and have completed at least upper secondary education, with more than one third holding a tertiary degree, while the proportion of tertiary educated is smaller among mothers than among childless women.

First and second birth transition rates

Overall, the first-birth transition rate equals 0.098, whilethe rate at which mothers of one child transition to a second birth is 0.071. We observe no significant difference in first-birth transition rates by the overall regional average of childcare usage from 2005 to 2018. Yet, mothers' second-birth transition rates are larger in regions where the overall level of CC usage higher: in regions in the highest quartile, the rate of transitioning to a second child is almost double than the rate estimated in all the other regions (S3 Table). First- and second-birth transition rates increase with mothers' educational level: the rates among tertiary educated women (0.113 and 0.0.93, respectively), are almost two times higher than those estimated among low-educated women (0.68 and 0.054, respectively). Employed women have a 32 percent higher rate of first-birth transition compared to inactive or unemployed women, while second-parity transition rates are similar between the two groups.

Tables

S1 Table. EU-SILC and public childcare usage data (ECEC).

Country	First EU-SILC	Last EU-SILC	EU-SILC NUTS level	First ECEC	Last ECEC	Inclusion
AT	2004	2020	NUTS-1	2000	2019	Included
BE	2004	2020	NUTS-1	2000	2019	Included
BG	2006	2020	NUTS-1/2	2000	2019	Included
CZ	2005	2020	NUTS-2	2001	2018	Included
DE	2015	2019	missing	2002	2018	Excluded
DK	2003	2020	NUTS-1	2000	2014	Excluded
ES	2004	2020	NUTS-2	2003	2019	Included
FI	2004	2020	NUTS-2	2000	2019	Included
FR	2004	2020	NUTS-2	2003	2019	Included
HR	2010	2020	NUTS-1	2003	2019	Excluded
HU	2004	2020	NUTS-1	2000	2019	Included
IE	2004	2020	NUTS-1	2015	2018	Excluded
IT	2004	2020	NUTS-1	2000	2020	Included
NL	2005	2020	missing	2007	2017	Excluded
NO	2003	2020	NUTS-1	2000	2019	Excluded
PL	2005	2020	NUTS-1	2000	2019	Included
RO	2007	2020	NUTS-1	2015	2018	Excluded
SE	2004	2020	NUTS-1	2000	2018	Included
SI	2005	2020	NUTS-1	2006	2019	Excluded
SK	2005	2020	NUTS-1	2001	2018	Excluded
UK (only ENG)	2005	2018	NUTS-1	2014	2019	Excluded

***S2 Table.* Distribution of individual-level variables and country of residence among childless women (left column) and mothers of one child (right column), measured at their first interview.**

	Childless women<br>n (%)	Mothers<br>n (%)
N	10,593	20,171
Age (5 year band)		
20-24	866 (8.2%)	588 (2.9%)
25-29	2,800 (26.4%)	2,853 (14.1%)
30-34	2,893 (27.3%)	5,324 (26.4%)
35-39	1,699 (16.0%)	4,840 (24.0%)
40-45	2,335 (22.0%)	6,566 (32.6%)
Civil status		
Not Married	5,237 (49.4%)	4,712 (23.4%)
Married	5,356 (50.6%)	15,459 (76.6%)
Educational level (3 cat.)		
Lower Secondary	1,170 (11.0%)	3,012 (14.9%)
Upper Secondary	4,645 (43.8%)	9,868 (48.9%)
Tertiary	4,778 (45.1%)	7,291 (36.1%)
Employment status (2 cat.)		
Unemployed-Inactive	2,374 (22.4%)	6,387 (31.7%)
Employed	8,219 (77.6%)	13,784 (68.3%)
Country		
Austria	790 (7.5%)	1,111 (5.5%)
Belgium	625 (5.9%)	921 (4.6%)
Bulgaria	292 (2.8%)	1,230 (6.1%)
Czech Republic	942 (8.9%)	2,005 (9.9%)
Spain	1,472 (13.9%)	2,828 (14.0%)
Finland	1,506 (14.2%)	1,445 (7.2%)
France	607 (5.7%)	1,009 (5.0%)
Hungary	732 (6.9%)	1,724 (8.5%)
Italy	1,731 (16.3%)	3,608 (17.9%)
Poland	1,124 (10.6%)	3,425 (17.0%)
Sweden	772 (7.3%)	865 (4.3%)

S3 Table. **Conditional probabilities (rates) of transitioning to a first or second birth by individual-level variables and area of residence, measured at their first interview.**

	Transition to parenthood	Second parity transition
Overall	0.0975	0.0705
	[0.898,0.907]	[0.926,0.933]
Age (5 year band)		
20-24	0.0617	0.0682
	[0.0512,0.0721]	[0.0541,0.0824]
25-29	0.0994	0.0929
	[0.0912,0.108]	[0.0840,0.102]
30-34	0.141	0.101
	[0.131,0.151]	[0.0945,0.108]
35-39	0.103	0.0796
	[0.0913,0.114]	[0.0736,0.0855]
40-45	0.0263	0.0212
	[0.020,0.032]	[0.017,0.026]
Civil status		
Not Married	0.0769	0.0813
	[0.071,0.083]	[0.074,0.089]
Married	0.116	0.0676
	[0.109,0.123]	[0.064,0.071]
Educational level (3 cat.)		
Lower Secondary	0.069	0.054
	[0.057,0.080]	[0.048,0.060]
Upper Secondary	0.090	0.061
	[0.083,0.096]	[0.056,0.065]
Tertiary	0.112	0.093
	[0.105,0.119]	[0.087,0.098]
Employment status (2 cat.)		
Unemployed-Inactive	0.079	0.074
	[0.070,0.087]	[0.069,0.079]
Employed	0.103	0.069
	[0.098,0.109]	[0.065,0.073]
By regional average of childcare usage in t-2		
1st quartile	0.111	0.064
	[0.101,0.121]	[0.060,0.068]
2nd quartile	0.097	0.069
	[0.086,0.108]	[0.062,0.076]
3rd quartile	0.091	0.063
	[0.084,0.098]	[0.059,0.068]
4th quartile	0.097	0.105
	[0.087,0.107]	[0.092,0.118]
Observations	35894	66696
Subjects	10593	20171

95% confidence intervals in squared brackets.

S4 Table. **Regression coefficients (Hazard Ratios) between a unit increase in ECEC and first birth transitions.**

	First parity Transition			
	M1	*M2*	*M3*	*M4*
	Overall	Education level interaction	Employment status interaction	Overall ECEC interaction
Demeaned ECEC in t-2	**1.026*****	**1.057*****	**0.989**	**1.078*****
	[1.011,1.042]	[1.016,1.100]	[0.960,1.018]	[1.029,1.130]
Interactions				
Upper Secondary X **Demeaned ECEC in t-2**		**0.965**		
		[0.920,1.011]		
Tertiary X **Demeaned ECEC in t-2**		**0.967**		
		[0.928,1.009]		
Employed X **Demeaned ECEC in t-2**			**1.048*****	
			[1.015,1.082]	
Regional average ECEC				0.993
				[0.975,1.012]
Regional average ECEC X **Demeaned ECEC in t-2**				**0.997***
				[0.994,1.000]
Regional average ECEC^2				1.000
				[1.000,1.000]
Regional average ECEC^2 X **Demeaned ECEC in t-2**				**1.000**
				[1.000,1.000]
Age group (ref. 20-24)				
25-29	1.345***	1.400***	1.364***	1.350***
	[1.104,1.638]	[1.148,1.707]	[1.121,1.659]	[1.108,1.644]
30-34	1.776***	1.889***	1.787***	1.803***
	[1.453,2.172]	[1.545,2.310]	[1.463,2.182]	[1.474,2.206]
35-39	1.246**	1.326**	1.251**	1.242*
	[1.002,1.550]	[1.065,1.651]	[1.008,1.554]	[0.999,1.545]
40-45	0.302***	0.319***	0.302***	0.302***
	[0.225,0.405]	[0.238,0.430]	[0.225,0.404]	[0.225,0.406]
Marital Status(ref. Not Married)				
Married	1.824***	1.801***	1.837***	1.833***
	[1.627,2.045]	[1.608,2.016]	[1.638,2.060]	[1.634,2.056]
Education level (ref. Lower Secondary)				
Upper Secondary	1.238**	1.322***	1.243**	1.236**
	[1.018,1.507]	[1.095,1.596]	[1.024,1.508]	[1.016,1.505]
Tertiary	1.426***	1.544***	1.427***	1.418***
	[1.178,1.726]	[1.284,1.855]	[1.182,1.724]	[1.169,1.719]
Employment status (ref. Not employed)				
Employed	1.237***		1.178***	1.217***
	[1.084,1.411]		[1.042,1.332]	[1.069,1.386]
Year of first interview (ref. 2010)				
2005	0.677***	0.686***	0.680***	0.716***
	[0.545,0.842]	[0.552,0.853]	[0.547,0.846]	[0.576,0.891]
2006	0.890	0.894	0.880	0.912
	[0.733,1.082]	[0.735,1.086]	[0.723,1.070]	[0.752,1.107]
2007	0.882	0.891	0.886	0.900
	[0.725,1.073]	[0.733,1.084]	[0.728,1.078]	[0.740,1.093]
2008	0.910	0.921	0.910	0.904
	[0.745,1.111]	[0.754,1.124]	[0.745,1.112]	[0.740,1.104]
2009	0.907	0.916	0.908	0.915
	[0.741,1.111]	[0.748,1.123]	[0.742,1.113]	[0.748,1.118]
2011	0.842	0.850	0.842	0.822*
	[0.681,1.041]	[0.687,1.051]	[0.680,1.041]	[0.666,1.015]
2012	0.884	0.882	0.883	0.872
	[0.712,1.097]	[0.710,1.097]	[0.710,1.097]	[0.703,1.082]

	2013	0.875	0.869	0.876	0.866
		[0.702,1.090]	[0.697,1.083]	[0.703,1.092]	[0.697,1.076]
	2014	0.899	0.895	0.901	0.884
		[0.723,1.118]	[0.720,1.114]	[0.724,1.121]	[0.712,1.099]
	2015	0.868	0.875	0.862	0.858
		[0.680,1.108]	[0.686,1.115]	[0.675,1.100]	[0.672,1.096]
	2016	0.764**	0.766**	0.762**	0.754**
		[0.591,0.987]	[0.592,0.991]	[0.589,0.986]	[0.581,0.977]
	2017	0.812	0.820	0.809	0.835
		[0.604,1.090]	[0.611,1.100]	[0.603,1.085]	[0.619,1.126]
Region of residence (ref. AT1)					
	AT2	1.385	1.375	1.423	
		[0.894,2.144]	[0.892,2.118]	[0.916,2.212]	
	AT3	1.224	1.216	1.264	
		[0.866,1.731]	[0.858,1.723]	[0.898,1.780]	
	BE1	1.106	1.077	1.136	
		[0.795,1.538]	[0.777,1.494]	[0.817,1.578]	
	BE2	1.358**	1.368**	1.408**	
		[1.009,1.828]	[1.019,1.837]	[1.047,1.892]	
	BG3	0.923	0.912	0.942	
		[0.562,1.515]	[0.558,1.489]	[0.577,1.537]	
	BG4	0.496**	0.482**	0.505**	
		[0.267,0.921]	[0.257,0.903]	[0.270,0.944]	
	CZ01	0.667*	0.659*	0.690*	
		[0.431,1.032]	[0.426,1.019]	[0.447,1.068]	
	CZ02	1.195	1.219	1.241	
		[0.795,1.797]	[0.811,1.832]	[0.826,1.863]	
	CZ03	0.893	0.912	0.925	
		[0.544,1.466]	[0.557,1.494]	[0.564,1.516]	
	CZ04	1.033	1.047	1.066	
		[0.629,1.696]	[0.638,1.719]	[0.650,1.748]	
	CZ05	1.486**	1.499**	1.537**	
		[1.011,2.184]	[1.019,2.203]	[1.047,2.256]	
	CZ06	1.393*	1.399*	1.447*	
		[0.960,2.022]	[0.966,2.027]	[0.998,2.097]	
	CZ07	1.666**	1.689**	1.724**	
		[1.094,2.536]	[1.110,2.570]	[1.133,2.621]	
	CZ08	1.524**	1.522**	1.570**	
		[1.042,2.229]	[1.040,2.229]	[1.075,2.294]	
	ES11	1.136	1.125	1.175	
		[0.718,1.796]	[0.710,1.781]	[0.742,1.859]	
	ES12	0.937	0.932	0.952	
		[0.557,1.575]	[0.550,1.578]	[0.568,1.596]	
	ES13	1.356	1.319	1.383	
		[0.821,2.239]	[0.794,2.194]	[0.839,2.281]	
	ES21	1.273	1.276	1.316	
		[0.858,1.887]	[0.862,1.890]	[0.887,1.953]	
	ES23	1.076	1.066	1.141	
		[0.644,1.796]	[0.626,1.816]	[0.692,1.882]	
	ES24	0.768	0.782	0.791	
		[0.435,1.354]	[0.445,1.374]	[0.445,1.408]	
	ES30	0.936	0.926	0.976	
		[0.633,1.385]	[0.627,1.367]	[0.659,1.445]	
	ES41	0.944	0.936	0.983	
		[0.582,1.531]	[0.579,1.514]	[0.607,1.592]	
	ES42	1.328	1.299	1.374	
		[0.870,2.026]	[0.854,1.976]	[0.901,2.096]	
	ES43	0.778	0.804	0.767	
		[0.393,1.543]	[0.411,1.571]	[0.378,1.557]	

ES51	1.064	1.060	1.101
	[0.737,1.536]	[0.732,1.534]	[0.763,1.587]
ES52	0.822	0.821	0.847
	[0.560,1.206]	[0.560,1.204]	[0.577,1.244]
ES53	0.664	0.661	0.689
	[0.356,1.238]	[0.359,1.219]	[0.366,1.298]
ES61	1.160	1.135	1.174
	[0.786,1.713]	[0.776,1.659]	[0.790,1.742]
ES62	1.427	1.411	1.478
	[0.677,3.009]	[0.656,3.038]	[0.700,3.123]
ES63	3.638***	3.300***	4.061***
	[1.939,6.823]	[1.827,5.962]	[2.075,7.949]
ES64	1.221	1.176	1.295
	[0.427,3.495]	[0.413,3.354]	[0.449,3.741]
ES70	1.260	1.269	1.286
	[0.500,3.170]	[0.507,3.177]	[0.512,3.227]
FI18	1.007	0.988	1.040
	[0.757,1.340]	[0.744,1.312]	[0.782,1.382]
FI19	1.093	1.067	1.128
	[0.787,1.518]	[0.769,1.480]	[0.814,1.564]
FI1D	1.033	1.001	1.066
	[0.732,1.456]	[0.711,1.410]	[0.757,1.501]
FR1	1.250	1.231	1.315
	[0.666,2.347]	[0.650,2.330]	[0.705,2.451]
FR2	0.630	0.584	0.626
	[0.332,1.196]	[0.293,1.167]	[0.329,1.191]
FR3	0.689	0.685	0.739
	[0.291,1.635]	[0.282,1.668]	[0.343,1.594]
FR4	1.138	1.190	1.080
	[0.550,2.356]	[0.586,2.415]	[0.514,2.271]
FR5	1.643**	1.607*	1.786**
	[1.016,2.657]	[0.978,2.639]	[1.114,2.863]
FR6	0.871	0.824	1.002
	[0.436,1.740]	[0.407,1.668]	[0.514,1.955]
FR7	1.760**	1.712**	1.759**
	[1.051,2.948]	[1.028,2.851]	[1.056,2.931]
FR8	1.659	1.618	1.728
	[0.805,3.417]	[0.795,3.293]	[0.817,3.655]
HU1	0.780	0.783	0.805
	[0.498,1.221]	[0.500,1.224]	[0.514,1.260]
HU2	1.106	1.112	1.146
	[0.731,1.673]	[0.734,1.685]	[0.758,1.734]
HU3	1.133	1.130	1.170
	[0.808,1.590]	[0.806,1.584]	[0.835,1.639]
ITC	1.077	1.091	1.115
	[0.798,1.454]	[0.809,1.471]	[0.827,1.504]
ITD	1.038	1.034	1.068
	[0.767,1.404]	[0.765,1.399]	[0.790,1.445]
ITE	0.988	0.962	1.016
	[0.724,1.350]	[0.704,1.313]	[0.745,1.387]
ITF	1.195	1.135	1.219
	[0.836,1.709]	[0.798,1.613]	[0.854,1.739]
ITG	1.254	1.174	1.276
	[0.809,1.945]	[0.759,1.814]	[0.823,1.976]
PL2	1.001	0.999	1.034
	[0.708,1.417]	[0.707,1.412]	[0.731,1.462]
PL4	1.187	1.195	1.227
	[0.826,1.704]	[0.834,1.712]	[0.855,1.760]
PL5	1.106	1.116	1.145

	[0.711,1.721]	[0.718,1.734]	[0.737,1.779]	
PL6	0.877	0.861	0.901	
	[0.600,1.282]	[0.590,1.257]	[0.617,1.316]	
PLN	1.213	1.202	1.250	
	[0.903,1.630]	[0.897,1.611]	[0.932,1.678]	
SE1	1.228	1.198	1.272	
	[0.898,1.679]	[0.878,1.634]	[0.931,1.737]	
SE2	1.315*	1.290	1.361*	
	[0.964,1.794]	[0.948,1.757]	[0.999,1.854]	
SE3	1.216	1.194	1.259	
	[0.807,1.832]	[0.793,1.797]	[0.836,1.897]	
Country of residence (ref. Austria)				
Belgium				1.139
				[0.914,1.420]
Bulgaria				0.612**
				[0.415,0.903]
Czech Republic				1.002
				[0.759,1.323]
Spain				0.889
				[0.722,1.095]
Finland				0.945
				[0.763,1.171]
France				1.244
				[0.675,2.293]
Hungary				0.854
				[0.660,1.104]
Italy				0.949
				[0.781,1.154]
Poland				0.918
				[0.675,1.248]
Sweden				1.079
				[0.631,1.848]
Person-years	*35894*	*35894*	*35894*	*35894*
Individuals	*10593*	*10593*	*10593*	*10593*

The exponential of the coefficient (Hazard Ratios) results from four *cloglog* regressions and 95% confidence in squared brackets. * $p < 0.10$, ** $p < 0.05$, *** $p < 0.01$

***S5 Table.* Regression coefficients (Hazard Ratios) between a unit increase in ECEC and second birth transitions.**

	Second birth transition			
	M1	*M2*	*M3*	*M4*
	Overall	Education level interaction	Employment status interaction	Overall ECEC interaction
Demeaned ECEC in t-2	**1.028*****	**1.020**	**1.025****	**1.062*****
	[1.015,1.042]	[0.986,1.055]	[1.001,1.049]	[1.024,1.101]
Interactions				
Upper Secondary X **Demeaned ECEC in t-2**		**1.026**		
		[0.978,1.077]		
Tertiary X **Demeaned ECEC in t-2**		**1.002**		
		[0.968,1.037]		
Employed X **Demeaned ECEC in t-2**			**1.005**	
			[0.978,1.034]	
Regional average ECEC				1.016**
				[1.001,1.032]
Regional average ECEC X **Demeaned ECEC in t-2**				**0.999**
				[0.996,1.001]
Regional average ECEC^2				1.000*
				[1.000,1.000]
Regional average ECEC^2 X **Demeaned ECEC in t-2**				**1.000**
				[1.000,1.000]
Age group (ref. 20-24)				
25-29	1.246*	1.209	1.247*	1.224
	[0.962,1.613]	[0.936,1.561]	[0.965,1.613]	[0.955,1.568]
30-34	1.241*	1.185	1.242*	1.226*
	[0.968,1.590]	[0.927,1.514]	[0.970,1.590]	[0.970,1.550]
35-39	0.965	0.917	0.966	0.949
	[0.750,1.242]	[0.715,1.175]	[0.751,1.242]	[0.747,1.206]
40-45	0.255***	0.240***	0.255***	0.249***
	[0.187,0.348]	[0.177,0.326]	[0.187,0.348]	[0.183,0.340]
Marital Status(ref. Not Married)				
Married	1.109*	1.120*	1.110*	1.131**
	[0.987,1.246]	[0.997,1.259]	[0.988,1.247]	[1.002,1.276]
Education level (ref. Lower Secondary)				
Upper Secondary	1.146*	1.085	1.146*	1.130
	[0.978,1.343]	[0.947,1.243]	[0.977,1.343]	[0.972,1.315]
Tertiary	1.561***	1.504***	1.560***	1.546***
	[1.346,1.812]	[1.315,1.719]	[1.344,1.810]	[1.336,1.790]
Employment status (ref. Not employed)				
Employed	0.865***		0.860***	0.874***
	[0.790,0.948]		[0.791,0.935]	[0.798,0.957]
Year of first interview (ref. 2010)				
2005	0.800**	0.806**	0.798**	0.832*
	[0.663,0.965]	[0.667,0.972]	[0.662,0.964]	[0.690,1.004]
2006	0.820**	0.826**	0.819**	0.846*
	[0.687,0.979]	[0.692,0.987]	[0.686,0.978]	[0.709,1.010]
2007	0.993	0.992	0.993	1.014
	[0.831,1.186]	[0.830,1.186]	[0.831,1.186]	[0.849,1.212]
2008	1.018	1.018	1.018	1.040
	[0.851,1.219]	[0.851,1.218]	[0.851,1.219]	[0.870,1.243]
2009	0.992	0.989	0.992	1.000
	[0.831,1.185]	[0.828,1.182]	[0.831,1.185]	[0.838,1.193]
2011	0.963	0.965	0.964	0.955
	[0.799,1.161]	[0.800,1.163]	[0.799,1.162]	[0.794,1.149]
2012	1.066	1.068	1.067	1.065
	[0.888,1.281]	[0.890,1.282]	[0.888,1.281]	[0.888,1.276]

	2013	0.975	0.975	0.975	0.968
		[0.804,1.181]	[0.805,1.182]	[0.804,1.181]	[0.800,1.172]
	2014	1.025	1.025	1.026	1.022
		[0.845,1.243]	[0.845,1.242]	[0.846,1.244]	[0.843,1.239]
	2015	1.091	1.088	1.091	1.077
		[0.874,1.362]	[0.873,1.357]	[0.874,1.361]	[0.862,1.344]
	2016	1.253**	1.257**	1.254**	1.247*
		[1.002,1.567]	[1.007,1.569]	[1.003,1.568]	[0.995,1.562]
	2017	1.096	1.093	1.094	1.117
		[0.866,1.387]	[0.865,1.382]	[0.864,1.385]	[0.886,1.408]
Region of residence (ref. AT1)					
	AT2	0.986	0.975	0.986	
		[0.680,1.431]	[0.671,1.416]	[0.680,1.430]	
	AT3	1.311*	1.296*	1.311*	
		[0.990,1.738]	[0.975,1.721]	[0.990,1.737]	
	BE1	1.262*	1.224	1.263*	
		[0.961,1.657]	[0.932,1.607]	[0.962,1.658]	
	BE2	1.254*	1.199	1.257*	
		[0.970,1.622]	[0.928,1.547]	[0.973,1.624]	
	BG3	0.425***	0.414***	0.426***	
		[0.297,0.610]	[0.289,0.594]	[0.297,0.610]	
	BG4	0.340***	0.331***	0.341***	
		[0.232,0.498]	[0.226,0.485]	[0.233,0.499]	
	CZ01	0.883	0.884	0.883	
		[0.605,1.287]	[0.608,1.286]	[0.605,1.287]	
	CZ02	0.856	0.844	0.857	
		[0.611,1.199]	[0.601,1.184]	[0.612,1.200]	
	CZ03	1.185	1.179	1.186	
		[0.881,1.594]	[0.875,1.588]	[0.882,1.594]	
	CZ04	0.762	0.756	0.762	
		[0.458,1.266]	[0.454,1.259]	[0.459,1.267]	
	CZ05	1.192	1.186	1.193	
		[0.858,1.655]	[0.853,1.648]	[0.859,1.656]	
	CZ06	1.381**	1.370**	1.382**	
		[1.013,1.882]	[1.003,1.869]	[1.014,1.882]	
	CZ07	0.976	0.963	0.977	
		[0.673,1.417]	[0.663,1.399]	[0.674,1.417]	
	CZ08	1.022	1.026	1.022	
		[0.734,1.424]	[0.737,1.430]	[0.734,1.423]	
	ES11	0.679*	0.658*	0.679*	
		[0.439,1.051]	[0.426,1.016]	[0.438,1.050]	
	ES12	0.730	0.740	0.732	
		[0.394,1.352]	[0.399,1.372]	[0.395,1.355]	
	ES13	0.914	0.879	0.914	
		[0.562,1.486]	[0.539,1.431]	[0.562,1.487]	
	ES21	1.402**	1.354*	1.405**	
		[1.014,1.939]	[0.979,1.872]	[1.016,1.942]	
	ES23	0.888	0.860	0.888	
		[0.513,1.538]	[0.494,1.498]	[0.514,1.534]	
	ES24	0.929	0.908	0.930	
		[0.563,1.533]	[0.551,1.498]	[0.564,1.535]	
	ES30	1.130	1.094	1.132	
		[0.797,1.600]	[0.773,1.548]	[0.800,1.602]	
	ES41	1.049	1.024	1.049	
		[0.700,1.571]	[0.684,1.534]	[0.701,1.571]	
	ES42	1.002	0.989	1.005	
		[0.674,1.491]	[0.666,1.469]	[0.676,1.495]	
	ES43	0.995	0.980	0.993	
		[0.586,1.691]	[0.583,1.650]	[0.583,1.690]	

ES51	1.156	1.112	1.159
	[0.817,1.636]	[0.786,1.573]	[0.819,1.640]
ES52	1.153	1.113	1.155
	[0.787,1.689]	[0.759,1.632]	[0.789,1.692]
ES53	1.157	1.123	1.161
	[0.694,1.931]	[0.673,1.873]	[0.696,1.935]
ES61	1.250	1.209	1.250
	[0.924,1.691]	[0.895,1.634]	[0.925,1.690]
ES62	1.097	1.052	1.098
	[0.701,1.717]	[0.673,1.645]	[0.702,1.718]
ES63	1.446	1.392	1.445
	[0.723,2.893]	[0.698,2.776]	[0.723,2.887]
ES64	1.310	1.308	1.311
	[0.612,2.804]	[0.605,2.826]	[0.612,2.808]
ES70	0.956	0.936	0.956
	[0.539,1.698]	[0.528,1.661]	[0.539,1.697]
FI18	1.595***	1.573***	1.596***
	[1.241,2.050]	[1.224,2.022]	[1.242,2.050]
FI19	1.381**	1.352**	1.382**
	[1.026,1.859]	[1.001,1.824]	[1.028,1.859]
FI1D	1.509***	1.499***	1.510***
	[1.121,2.032]	[1.112,2.020]	[1.122,2.032]
FR1	1.664*	1.635*	1.665*
	[0.934,2.965]	[0.923,2.898]	[0.935,2.964]
FR2	0.657	0.639*	0.650
	[0.386,1.117]	[0.376,1.086]	[0.378,1.118]
FR3	1.950	1.847	1.917
	[0.705,5.391]	[0.660,5.163]	[0.691,5.321]
FR4	0.738	0.674	0.734
	[0.246,2.213]	[0.245,1.856]	[0.247,2.183]
FR5	0.942	0.900	0.939
	[0.534,1.660]	[0.506,1.602]	[0.534,1.650]
FR6	0.862	0.798	0.860
	[0.424,1.753]	[0.391,1.630]	[0.417,1.774]
FR7	0.623	0.570*	0.616
	[0.342,1.134]	[0.314,1.033]	[0.336,1.128]
FR8	0.594	0.569	0.594
	[0.282,1.253]	[0.270,1.200]	[0.282,1.252]
HU1	0.674*	0.661*	0.675*
	[0.442,1.028]	[0.432,1.012]	[0.443,1.028]
HU2	0.631**	0.620***	0.631**
	[0.444,0.897]	[0.436,0.883]	[0.444,0.897]
HU3	0.885	0.876	0.885
	[0.667,1.175]	[0.660,1.162]	[0.668,1.174]
ITC	1.026	1.000	1.027
	[0.779,1.351]	[0.760,1.316]	[0.780,1.352]
ITD	0.996	0.968	0.996
	[0.763,1.300]	[0.742,1.263]	[0.764,1.300]
ITE	0.953	0.942	0.954
	[0.724,1.255]	[0.716,1.240]	[0.725,1.255]
ITF	0.818	0.830	0.817
	[0.597,1.121]	[0.606,1.135]	[0.596,1.119]
ITG	0.681**	0.686**	0.681**
	[0.468,0.992]	[0.472,0.997]	[0.468,0.990]
PL2	0.698**	0.686**	0.699**
	[0.519,0.940]	[0.510,0.922]	[0.520,0.940]
PL4	0.887	0.860	0.888
	[0.666,1.183]	[0.645,1.147]	[0.667,1.184]
PL5	0.624**	0.609***	0.624**

	[0.430,0.904]	[0.421,0.883]	[0.431,0.904]	
PL6	0.853	0.829	0.854	
	[0.635,1.144]	[0.617,1.112]	[0.637,1.145]	
PLN	0.923	0.893	0.925	
	[0.726,1.174]	[0.702,1.135]	[0.728,1.175]	
SE1	1.715***	1.638***	1.719***	
	[1.308,2.248]	[1.252,2.144]	[1.312,2.251]	
SE2	1.675***	1.601***	1.678***	
	[1.293,2.169]	[1.237,2.071]	[1.297,2.172]	
SE3	1.935***	1.850***	1.939***	
	[1.405,2.665]	[1.347,2.543]	[1.410,2.668]	
Country of residence (ref. Austria)				
Belgium				1.119
				[0.928,1.349]
Bulgaria				0.362***
				[0.279,0.469]
Czech Republic				1.087
				[0.873,1.354]
Spain				1.027
				[0.862,1.223]
Finland				1.337***
				[1.110,1.610]
France				1.194
				[0.668,2.133]
Hungary				0.711***
				[0.575,0.878]
Italy				0.874
				[0.737,1.035]
Poland				0.914
				[0.715,1.168]
Sweden				1.638*
				[0.998,2.689]
Person-years	*66696*	*66696*	*66696*	*66696*
Individuals	*20171*	*20171*	*20171*	*20171*

The exponential of the coefficient (Hazard Ratios) results from four *cloglog* regressions and 95% confidence in squared brackets. * $p < 0.10$, ** $p < 0.05$, *** $p < 0.01$
