# Supplementary material for: Early public childcare and fertility: A longitudinal study for Europe
Source: PLoS One. 2026 Jul 14;21(7):e0353502. doi: 10.1371/journal.pone.0353502 (PMC13367713; doi:10.1371/journal.pone.0353502)
Supplement: Attachment — Submitted filename: Response to Reviewers.docx [file pone.0353502.s006.docx]

Response to reviewers

Dear Editor and Reviewers,

We are sincerely grateful for the reviewers’ constructive comments and insightful suggestions and the opportunity to represent this paper. In light of these comments, we have carefully revised the submitted manuscript. First, we have more explicitly acknowledged the limitations of ECEC usage as a proxy measure in the Data sub-section and the Limitations paragraph. This addresses a concern raised by both reviewers regarding the operationalization and interpretation of the main explanatory variable. Second, we have translated the initially presented relative associations into absolute transition *rates* to aid policy interpretation. Third, we have clarified the scope of the regional fixed-effects strategy, explicitly acknowledging that region and year dummies cannot account for region-specific time-varying confounders. Fourth, we have reframed the 22% ECEC threshold as an indicative, sample-specific benchmark rather than a universal policy target. In addition, all minor points raised by both reviewers have been considered and addressed, with corresponding edits made throughout the manuscript. Reponses and changes in the revised manuscript are shown in blue; line numbers refer to the revised version.

**Response to Reviewer 1’s comments**

We sincerely thank Reviewer 1 for the thoughtful and constructive comments on our manuscript. Based on these comments, we have carefully revised the original manuscript. Please find below our responses and revisions.

**Comment 1: Using ECEC as a Proxy**

When childcare usage is used as a proxy for availability, policy supply is confused with eligibility requirements, prevailing norms, and parental demand. The estimated associations may reflect broader behavioral or labor-market changes rather than just childcare capacity, and this should be more clearly acknowledged in the manuscript.

Yes. We agree that the association between childcare usage and fertility might reflect not only change in availability or eligibility requirements (already noted in the discussion) but also in the demand side through, e.g., changes in parental norms and in the labor market. In the revised manuscript, the limitations of this proxy measure are now more explicitly acknowledged in the Data sub-section (line 154) and in the Discussion sections when presenting the study’s limitations (line 410). The corresponding changes in the revised main text are as follows:

Line 154: “[…] Actual enrolment, however, also serves as a proxy for availability given the general undersupply of public early childcare across European regions."

Lines 406: “Moreover, childcare usage might reflect not only the supply-side capacity and eligibility rules, but also a combination of prevailing gender norms around infant childcare enrolment, labor market changes, and parental preferences, meaning that the estimated associations may partly capture broader institutional and societal changes rather than childcare availability alone.”

**Comment 2: Confounding that Changes Over Time**

Although regional demeaning accounts for stable regional differences, it is unable to take into consideration time-varying unobserved factors, such as concurrent changes in family policy or changing employment conditions. This restriction calls for a more thorough explanation.

Yes. Our models include region-of-residence dummies and year-of-entry dummies that, together, absorb regional heterogeneity and account for common trends. The two-year lag on ECEC further reduces reverse causality concerns. However, region-specific time-varying unobserved factors, such as concurrent regional changes in the labor market or regional family policies other than ECEC, cannot be ruled out with the current design. Most policy changes, such as leaves or benefits, take place on the national level, though, that we can account for. We have clarified, in the Materials and Methods section, that region dummies and year dummies account for time-stable regional heterogeneity and a common time trend, separately, and explicitly acknowledged that our design cannot rule out region-specific time-varying unobserved factors, as a limitation. Specifically, we edited the manuscript as follows:

Line 186: “Region-of-residence dummies absorb time-stable differences across regions in fertility behavior, labor market structure, and family policy context, while year-of-entry dummies account for common temporal trends affecting all regions simultaneously. Together, they reduce concerns about confounding by stable compositional differences and aggregate period shocks. However, this strategy cannot account for region-specific time-varying unobserved factors, such as concurrent regional changes in labor market conditions or family support policies other than ECEC expansion.”

Line 410: “Additionally, although region and year dummies absorb time-stable regional heterogeneity and a common time trend, they cannot account for region-specific time-varying unobserved confounders, such as regional shifts in labor market conditions, parental leave provision, or other family policies.”

**Comment 3: Short Panel and Interpretation of Fertility**

Inference is limited to short-term parity transitions by the four-year EU-SILC panel. It is best to frame claims about a decrease in the difference between intended and actual fertility as suggestive rather than definitive.

We agree and thank the reviewer for raising this important comment. The four-year EU-SILC panel limits inference to short-term parity transitions, and effects on fertility should be framed as suggestive rather than definitive. We have revised the manuscript accordingly in the Abstract and the Discussion sections, as follows:

Line 26: "Our results suggest that investing in public childcare is associated, in the short term, with reducing the gap between intended and actual fertility in Europe, particularly in contexts where provision is lower.

Line 373: “What is more, childcare expansions might help in reducing the mismatch between desired and actual fertility, as indicated by higher short-term parity transition rates. Whether these associations persist and translate into higher completed fertility calls for future research employing longer follow-up windows.”

Lines 393: "Second, the short panel framework provided by EU-SILC comes with drawbacks stemming from left and right censoring, limiting our analysis to short-term parity transitions."

**Comment 4: Absolute versus Relative Effects**

Given the low baseline transition rates, large relative effects translate into modest absolute changes. More focus on absolute effect sizes would be beneficial for the policy debate in particular

We sincerely appreciate this suggestion. Focusing on absolute changes in transition rates is indeed more useful for the current policy debate, while acknowledging that baseline transition rates are small over the four-year panel. We have then translated relative associations in absolute increases in the Results and Discussion sections. The corresponding changes in the revised main text are as follows:

Line 246: “This translates to an increase in transition rates from 9.8 to 12.2 first births per 100 women and from 7.1 to 9.0 second births.”

Line 369: “[…] according to our findings, an increase of 10% in the share of children in public childcare is associated with an increase in first-birth transition rates from 9.8 to 12.2 births per 100 women, and from 7.1 to 9.0 second births, in the following four years – equivalent to about a one-third increase in transition rates.”

**Comment 5: Effects of Saturation**

A significant contribution is the identification of thresholds beyond which ECEC expansion exhibits no correlation with fertility; however, these cut-points are model-dependent and empirically derived. They shouldn't be offered as exact standards for policy.

We agree. The ECEC threshold of 22%, above which we find no association between ECEC increase and fertility is model and data dependent. The empirical distributional values (quartiles) calculated on the administrative childcare data itself, are presented in lines 292–294 of the original manuscript: "[…] 25% of regions in our sample reporting an overall average ECEC below 6%, while 50% are below 12%, and 75% are below 23%." Therefore, our findings show that the association between ECEC expansion and fertility is significant below this quartile boundary (i.e., in the bottom three quarters of the European regional childcare distribution available in our analytical sample) and weakens in the top quartile where childcare is already relatively common. Yet, this threshold needs to be interpreted as an indicative, sample-specific benchmark rather than a universal policy standard, given that the analytical sample does not cover all EU regions and that existing policy targets are informed by broader criteria beyond the childcare-fertility association alone. We have then redacted the manuscript as follows:

Line 304: "In regions where public ECEC exceeds 22% overall (i.e., 75% of the regions in our sample), we find no statistically significant association […]."

Line 363: "These thresholds however reflect the distributional structure of our analytical sample and should be interpreted as indicative benchmarks rather than universal policy standards, given that existing policy targets are informed by broader criteria beyond the childcare-fertility association alone."

Line 426: “[…] especially in regions where said services are insufficient (i.e., three quarters of the regions in our data, that show an average childcare usage below 23% and have not departed from such threshold by 2019) […]."

**Small Points**

**The Own-Children Approach**

Although the robustness checks are comforting, transparency would be enhanced by a brief statement recognizing that residual misclassification may differ depending on socioeconomic status.

We have edited the manuscript to acknowledge the reviewer’s point in the limitations paragraph. While some methodological studies on the Own-Children Approach find no major systematic differences between social groups in the underestimation of fertility (Greulich and Dasré 2017; Krapf and Kreyenfeld 2015), other find, using French data, that the number of children living outside the household is not randomly distributed, pointing to socioeconomic differentials and a systematic bias in the measurement, where underestimation particularly affects lower-educated women and those with a migration background (Greulich and Dasré 2019).

Line 391: “Although misclassification bias in the own-children method is unlikely to bias fertility estimates substantially [32,51], residual misclassification might be unevenly distributed across socioeconomic groups [31].”

31. Greulich, Angela, and Aurélien Dasré. 2019. ‘Observing the Number of Children with EU-SILC: A Quantification of Biases’. Population Vol. 73(4):685–720. doi:10.3917/popu.1804.0719.

32. Greulich, Angela, and Aurélien Dasré. 2017. ‘The Quality of Periodic Fertility Measures in EU-SILC’. Demographic Research 36:525–56. doi:10.4054/DemRes.2017.36.17.

51. Krapf, Sandra, and Michaela R. Kreyenfeld. 2015. Fertility Assessment with the Own-Children Method: A Validation with Data from the German Mikrozensus. 0 edn. TR-2015-003. Rostock: Max Planck Institute for Demographic Research. doi:10.4054/MPIDR-TR-2015-003.

**Selecting a Country**

In addition to the assurance provided by the national-level robustness checks, the exclusion of nations without sub-national data should be more prominently acknowledged.

We have added a sentence explicitly acknowledging the exclusion of some countries due to the missing information on the sub-national region of residence in the Materials and methods section. Precisely, in line 128 we reviewed the manuscript as follows: “[…] and excluded countries lacking sub-national residence data in EU-SILC, which are listed in Supporting Information, Table S1. Sensitivity analyses confirm that including these countries using national-level childcare measures does not alter our main findings.”

**The Impact of Anticipation**

Although anticipation effects might not be captured, lagging ECEC lowers the possibility of reverse causality. The methodological discussion would be strengthened by a brief recognition of this possibility.

We acknowledge that lagging ECEC by two years, while helping to partially rule out reverse causality, may not capture anticipation effects. Therefore, we have updated the manuscript as follows:

Line 162: “To reduce the likelihood of reverse causality, ECEC is lagged by two years, although this does not, in principle, preclude anticipation effects, whereby couples may adjust fertility intentions in response to announced childcare expansions in later years.”

**Causal Terminology**

To ensure consistency with the associational framework used throughout, the discussion should be examined for any language that might be interpreted as suggesting causation.

We have scanned the manuscript for any residual causal-sounding verbs or phrases that slip through despite the overall associational framing, as explicitly disclaimed in lines 357–360 in the submitted manuscript. Precisely, we have revised the manuscript as follows:

Line 237: “One percentage point increase in ECEC is associated with an increase in first-birth rates of 0.24 points (AME=0.0024, P=0.001) and in second-birth rates of 0.19 points (AME=0.0019, P<0.001).”

Line 333: “These factors are likely pathways underlying the reported positive associations, which have been found to promote total fertility and reduce postponement”

Line 422: “Increasing the provision of early child education and care services instead not only invest explicitly in the future generation but is also associated with greater gender equality, which in turn has been linked with higher fertility.”

Line 425: “Overall, our results provide a clear picture of the role of publicly subsidized childcare in supporting transitions to parenthood and to second births, especially in regions where said services are insufficient […]”

**Response to Reviewer 2’s comments**

We sincerely thank Reviewer 2 for the kind words and the valuable suggestions concerning our manuscript. In response to these suggestions, we have revised the manuscript accordingly, and our detailed responses to the comments that suggested a revision are presented below in blue.

**Comment 1: Data and Methods**

Although information on how demand for ECEC is calculated is provided in the referred article, I suggest clarifying this further in the main text. Specifically, on page 7, line 151, it might be helpful to explain more explicitly how the main explanatory variable is defined (and later point out that the explanatory variable referring to demand is actually use). Please be very explicit about whether the outcome variable represents use, i.e., the number of 0–2-year-old children participating in early childhood education divided by the total number of children aged 0–2 in the region.

We agree and thank the reviewer for pointing this out. We made explicit the precise operationalization of our main variable of interest (ECEC) directly in the text rather than deferred to Scherer & Pavolini (2023). We then updated the manuscript as follows:

Line 150: “The main explanatory variable is the yearly regional rate of public early childcare (ECEC) usage for children aged 0–2, demeaned at the regional level and lagged by two years. This is defined as the number of children aged 0–2 enrolled in publicly subsidized childcare divided by the total number of children aged 0–2 in the region, and captures actual enrolment rather than the number of available slots. Actual enrolment, however, also serves as a proxy for availability given the general undersupply of public early childcare across European regions.”

**Comment 2: Small variation in some countries**

It may also be worth noting that in some countries the use of early childhood education and care services has developed more slowly than in others due to policy changes, particularly related to parental leave schemes and home care allowance (cash-for-care benefit). This may be the case, for example, in the Czech Republic, Finland, possibly Hungary, as well as other Central European countries where parental leave durations have been extended (see, e.g., Bičáková & Kalíšsková, 2019; Canaan et al., 2022, Österbacka & Räsänen, 2022).

These parental leave or cash-for-care policies have, to some extent, reduced the use of early childhood education among the youngest children, while use has increased among slightly older age groups (e.g., lower participation among 0–1-year-olds and increased participation among one- and two-year-olds and older children). This point could be briefly mentioned, for instance in the discussion section or on page 9, line 199 (with a maximum of 1 or 2 sentences, since this issues is outside the scope of the present research).

Canaan, S., Lassen, A. S., Rosenbaum, P., & Steingrimsdottir, H. (2022). Maternity Leave and Paternity Leave: Evidence on the Economic Impact of Legislative Changes in High-Income Countries. In Oxford Research Encyclopedia of Economics and Finance. https://doi.org/10.1093/acrefore/9780190625979.013.806

Bičáková, A., & Kalíšsková, K. (2019). (Un)intended effects of parental leave policies: Evidence from the Czech Republic. Labour Economics. https://doi.org/10.1016/j.labeco.2019.07.003

Österbacka, E., & Räsänen, T. (2022). Back to work or stay at home? Family policies and maternal employment in Finland. Journal of Population Economics, 35(3), 1071–1101. https://doi.org/10.1007/s00148-021-00843-4

We sincerely thank the reviewer for this comment on the implications of longer parental leave schemes and the reduced enrolment of younger children in early childhood education and care services. We have updated the revised manuscript accordingly in the first paragraph of the Results section and refer now to the suggested literature.

Line 211: “In the Czech Republic and Finland, the slow expansion of ECEC for children aged 0–2 may somewhat reflect the introduction of long parental leave schemes and cash-for-care benefits, which might keep mothers out of the labor market when public childcare is scarce or expensive (Olivetti and Petrongolo 2017), thus potentially reducing formal care use among infants (Bičáková and Kalíšková 2019; Österbacka and Räsänen 2022).”

Olivetti, Claudia, and Barbara Petrongolo. 2017. ‘The Economic Consequences of Family Policies: Lessons from a Century of Legislation in High-Income Countries’. Journal of Economic Perspectives 31(1):205–30. doi:10.1257/jep.31.1.205.

**Comment 3: Results**

The results are clearly presented using figures, which I find effective and appropriate. In the first row of Figure 3 (first childbirth), the result for unemployed individuals appears somewhat puzzling, as the average marginal effect seems to decline as lagged regional day care use increases. However, as I understand it, the coefficients at value –10 and value +10 are proximately the same and the difference between coefficients from -10 to +10 are statistically insignificant. This suggests that among individuals in a weaker labor market attachment, the expansion of early childhood education services is not associated with fertility outcomes. This is also an interesting finding in itself.

We agree on clearly reporting the lack of association between early childcare usage expansions and first birth transitions as a finding in itself. We thank the reviewer for their suggestion and have updated the manuscript as follows:

Line 288: “[…] an increase in ECEC is positively associated with first-birth transitions only among employed women (AME = 0.0034, P < 0.001), while no significant association is observed among non-employed women (AME = -0.0009, P = 0.450). The absence of an association among non-employed women for first-birth transitions suggests that, where labor market attachment is weak, ECEC expansions do not appear to lower the barriers to parenthood. ECEC expansions however are positively associated with second-birth transition rates […].”

We are grateful for the careful review and hope the revised manuscript fully addresses all concerns raised. We sincerely appreciate the time and effort you have dedicated to reviewing our work.
