# Supplementary material for: Early public childcare and fertility: A longitudinal study for Europe
Source: PLoS One. 2026 Jul 14;21(7):e0353502. doi: 10.1371/journal.pone.0353502 (PMC13367713; doi:10.1371/journal.pone.0353502)
Supplement: Attachment — Submitted filename: Review comments.docx [file pone.0353502.s004.docx]

In my view, the manuscript is well written: the analyses are sufficient, the research question is clearly motivated and the scope of the manuscript is “just right”. The authors also acknowledge potential limitations of the study in a transparent manner and are able to provide policy recommendations based on the results. Manuscripts of this level of quality are rarely submitted for review, and I am therefore pleased with the overall quality of the paper.

Below, I list a few minor comments and a few suggestions that the authors may wish to consider in order to further improve the manuscript. All comments provided are entirely optional.

**Abstract and Introduction**
In the introduction the research is clearly motivated, and I am particularly pleased with the clarity of the abstract.

**Data and Methods**
The data and methods are clearly described. The use of a GLM with a log link is appropriate and well justified. I consider it a particular strength that potential reverse causality is addressed by using a lagged variable.

Although information on how demand for ECEC is calculated is provided in the referred article, I suggest clarifying this further in the main text. Specifically, on **page 7, line 151**, it might be helpful to explain more explicitly how the main explanatory variable is defined (and later point out that the explanatory variable referring to demand is actually use). Please be very explicit about whether the outcome variable represents use, i.e., the number of 0–2-year-old children participating in early childhood education divided by the total number of children aged 0–2 in the region.

**Small variation in some countries**

It may also be worth noting that in some countries the use of early childhood education and care services has developed more slowly than in others due to policy changes, particularly related to parental leave schemes and home care allowance (cash-for-care benefit). This may be the case, for example, in the Czech Republic, Finland, possibly Hungary, as well as other Central European countries where parental leave durations have been extended (see, e.g., Bičáková & Kalíšsková, 2019; Canaan et al., 2022, Österbacka & Räsänen, 2022).

These parental leave or cash-for-care policies have, to some extent, reduced the use of early childhood education among the youngest children, while use has increased among slightly older age groups (e.g., lower participation among 0–1-year-olds and increased participation among one- and two-year-olds and older children). This point could be briefly mentioned, for instance in the **discussion section or on page 9, line 199** (with a maximum of 1 or 2 sentences, since this issues is outside the scope of the present research).

**Results**
The results are clearly presented using figures, which I find effective and appropriate. In the first row of Figure 3 (first childbirth), the result for unemployed individuals appears somewhat puzzling, as the average marginal effect seems to decline as lagged regional day care use increases. However, as I understand it, the coefficients at value –10 and value +10 are proximately the same and the difference between coefficients from -10 to +10 are statistically insignificant. This suggests that among individuals in a weaker labour market attachment, the expansion of early childhood education services is not associated with fertility outcomes. This is also an interesting finding in itself.

**Robustness**

I appreciate that the authors have carefully considered issues arising from the EU-SILC panel structure and have tested alternative models and methods.

**Conclusion**
I particularly appreciate the inclusion of policy recommendations. The main message—that expanding the availability of early childhood education services, especially in regions where access is currently limited, may support family formation and possibly improve work–life balance and promote female employment—is well articulated and policy relevant.

**References**

Canaan, S., Lassen, A. S., Rosenbaum, P., & Steingrimsdottir, H. (2022). Maternity Leave and Paternity Leave: Evidence on the Economic Impact of Legislative Changes in High-Income Countries. In Oxford Research Encyclopedia of Economics and Finance. <https://doi.org/10.1093/acrefore/9780190625979.013.806>

Bičáková, A., & Kalíšsková, K. (2019). (Un)intended effects of parental leave policies: Evidence from the Czech Republic. Labour Economics. <https://doi.org/10.1016/j.labeco.2019.07.003>

Österbacka, E., & Räsänen, T. (2022). Back to work or stay at home? Family policies and maternal employment in Finland. Journal of Population Economics, 35(3), 1071–1101. <https://doi.org/10.1007/s00148-021-00843-4>
