# Supplementary material for: Early public childcare and fertility: A longitudinal study for Europe
Source: PLoS One. 2026 Jul 14;21(7):e0353502. doi: 10.1371/journal.pone.0353502 (PMC13367713; doi:10.1371/journal.pone.0353502)
Supplement: Peer review history

# Peer review: Decision Letter 0

**DOI:** 10.1371/journal.pone.0353502.r001

**Authors:** Iacopo Odoardi

1 May 2026

PONE-D-26-07298

Early public childcare and fertility: A longitudinal study for Europe

PLOS One

Dear Dr. Minchio,

Thank you for submitting your manuscript to PLOS ONE. After careful consideration, we feel that it has merit but does not fully meet PLOS ONE’s publication criteria as it currently stands. Therefore, we invite you to submit a revised version of the manuscript that addresses the points raised during the review process.

Dear Authors, thank you for the opportunity to consider your research. Your article has been reviewed by two referees; below you will find a brief comment from me, together with the Referees' reports, which are also attached.

Please submit your revised manuscript by Jun 15 2026 11:59PM. If you will need more time than this to complete your revisions, please reply to this message or contact the journal office at plosone@plos.org. When you're ready to submit your revision, log on to https://www.editorialmanager.com/pone/ and select the 'Submissions Needing Revision' folder to locate your manuscript file.

Please include the following items when submitting your revised manuscript:

- A letter that responds to each point raised by the academic editor and reviewer(s). You should upload this letter as a separate file labeled 'Response to Reviewers'.
- A marked-up copy of your manuscript that highlights changes made to the original version. You should upload this as a separate file labeled 'Revised Manuscript with Track Changes'.
- An unmarked version of your revised paper without tracked changes. You should upload this as a separate file labeled 'Manuscript'.

If you would like to make changes to your financial disclosure, please include your updated statement in your cover letter. Guidelines for resubmitting your figure files are available below the reviewer comments at the end of this letter.

If applicable, we recommend that you deposit your laboratory protocols in protocols.io to enhance the reproducibility of your results. Protocols.io assigns your protocol its own identifier (DOI) so that it can be cited independently in the future. For instructions see: https://journals.plos.org/plosone/s/submission-guidelines#loc-laboratory-protocols. Additionally, PLOS ONE offers an option for publishing peer-reviewed Lab Protocol articles, which describe protocols hosted on protocols.io. Read more information on sharing protocols at https://plos.org/protocols?utm_medium=editorial-email&utm_source=authorletters&utm_campaign=protocols.

As the corresponding author, your ORCID iD is verified in the submission system and will appear in the published article. PLOS supports the use of ORCID, and we encourage all coauthors to register for an ORCID iD and use it as well. Please encourage your coauthors to verify their ORCID iD within the submission system before final acceptance, as unverified ORCID iDs will not appear in the published article. *Only*  the individual author can complete the verification step; PLOS staff *cannot*  verify ORCID iDs on behalf of authors.

We look forward to receiving your revised manuscript.

Kind regards,

Iacopo Odoardi, Ph.D.

Academic Editor

PLOS One

Journal Requirements:

When submitting your revision, we need you to address these additional requirements.

1. Please ensure that your manuscript meets PLOS ONE's style requirements, including those for file naming. The PLOS ONE style templates can be found at

https://journals.plos.org/plosone/s/file?id=wjVg/PLOSOne_formatting_sample_main_body.pdf and

https://journals.plos.org/plosone/s/file?id=ba62/PLOSOne_formatting_sample_title_authors_affiliations.pdf

2. We note that the grant information you provided in the ‘Funding Information’ and ‘Financial Disclosure’ sections do not match.

When you resubmit, please ensure that you provide the correct grant numbers for the awards you received for your study in the ‘Funding Information’ section.

3. Thank you for stating the following financial disclosure:

“G.M. acknowledges the support of the NRRP MUR program funded by the NextGenerationEU. Funded by the European Union – NextGenerationEU. Views and opinions expressed are however those of the authors only and do not necessarily reflect those of the European Union or the European Commission. Neither the European Union nor the European Commission can be held responsible for them.”

Please state what role the funders took in the study.  If the funders had no role, please state: "The funders had no role in study design, data collection and analysis, decision to publish, or preparation of the manuscript."

If this statement is not correct you must amend it as needed.

Please include this amended Role of Funder statement in your cover letter; we will change the online submission form on your behalf.

4. In the online submission form you indicate that your data is not available for proprietary reasons and have provided a contact point for accessing this data. Please note that your current contact point is a co-author on this manuscript. According to our Data Policy, the contact point must not be an author on the manuscript and must be an institutional contact, ideally not an individual. Please revise your data statement to a non-author institutional point of contact, such as a data access or ethics committee, and send this to us via return email. Please also include contact information for the third party organization, and please include the full citation of where the data can be found.

5. If the reviewer comments include a recommendation to cite specific previously published works, please review and evaluate these publications to determine whether they are relevant and should be cited. There is no requirement to cite these works unless the editor has indicated otherwise.

6. Please review your reference list to ensure that it is complete and correct. If you have cited papers that have been retracted, please include the rationale for doing so in the manuscript text, or remove these references and replace them with relevant current references. Any changes to the reference list should be mentioned in the rebuttal letter that accompanies your revised manuscript. If you need to cite a retracted article, indicate the article’s retracted status in the References list and also include a citation and full reference for the retraction notice.

Additional Editor Comments:

Dear Authors, I am pleased to inform you that both Reviewers have found your research to be interesting and, overall, consider it to be sufficiently complete and detailed, and close to the standard required for publication. However, they have provided a number of comments and suggestions, which I ask you to consider carefully and to incorporate into your manuscript. Some of these points require particular attention and will certainly help improve both the quality of the research and its overall presentation.

[Note: HTML markup is below. Please do not edit.]

Reviewers' comments:

Reviewer's Responses to Questions

**Comments to the Author**

1. Is the manuscript technically sound, and do the data support the conclusions?

The manuscript must describe a technically sound piece of scientific research with data that supports the conclusions. Experiments must have been conducted rigorously, with appropriate controls, replication, and sample sizes. The conclusions must be drawn appropriately based on the data presented.

Reviewer #1: Yes

Reviewer #2: Yes

**********

2. Has the statistical analysis been performed appropriately and rigorously?

Reviewer #1: Yes

Reviewer #2: Yes

**********

3. Have the authors made all data underlying the findings in their manuscript fully available?

The PLOS Data policy requires authors to make all data underlying the findings described in their manuscript fully available without restriction, with rare exception (please refer to the Data Availability Statement in the manuscript PDF file). The data should be provided as part of the manuscript or its supporting information, or deposited to a public repository. For example, in addition to summary statistics, the data points behind means, medians and variance measures should be available. If there are restrictions on publicly sharing data—e.g. participant privacy or use of data from a third party—those must be specified.

Reviewer #1: Yes

Reviewer #2: Yes

**********

4. Is the manuscript presented in an intelligible fashion and written in standard English?

PLOS ONE does not copyedit accepted manuscripts, so the language in submitted articles must be clear, correct, and unambiguous. Any typographical or grammatical errors should be corrected at revision, so please note any specific errors here.

Reviewer #1: Yes

Reviewer #2: Yes

**********

5. Review Comments to the Author

Please use the space provided to explain your answers to the questions above. You may also include additional comments for the author, including concerns about dual publication, research ethics, or publication ethics. (Please upload your review as an attachment if it exceeds 20,000 characters)

Reviewer #1: 1. Using ECEC as a Proxy

When childcare usage is used as a proxy for availability, policy supply is confused with eligibility requirements, prevailing norms, and parental demand. The estimated associations may reflect broader behavioural or labour-market changes rather than just childcare capacity, and this should be more clearly acknowledged in the manuscript.

2. Confounding that Changes Over Time

Although regional demeaning accounts for stable regional differences, it is unable to take into consideration time-varying unobserved factors, such as concurrent changes in family policy or changing employment conditions. This restriction calls for a more thorough explanation.

3. Short Panel and Interpretation of Fertility

Inference is limited to short-term parity transitions by the four-year EU-SILC panel. It is best to frame claims about a decrease in the difference between intended and actual fertility as suggestive rather than definitive.

4. Absolute versus Relative Effects

Given the low baseline transition rates, large relative effects translate into modest absolute changes. More focus on absolute effect sizes would be beneficial for the policy debate in particular.

5. Effects of Saturation

A significant contribution is the identification of thresholds beyond which ECEC expansion exhibits no correlation with fertility; however, these cut-points are model-dependent and empirically derived. They shouldn't be offered as exact standards for policy.

Small Points

1. The Own-Children Approach

Although the robustness checks are comforting, transparency would be enhanced by a brief statement recognising that residual misclassification may differ depending on socioeconomic status.

2. Selecting a Country

In addition to the assurance provided by the national-level robustness checks, the exclusion of nations without sub-national data should be more prominently acknowledged.

3. The Impact of Anticipation

Although anticipation effects might not be captured, lagging ECEC lowers the possibility of reverse causality. The methodological discussion would be strengthened by a brief recognition of this possibility.

4. Causal Terminology

To ensure consistency with the associational framework used throughout, the discussion should be examined for any language that might be interpreted as suggesting causation.

Reviewer #2: In my view, the manuscript is well written: the analyses are sufficient, the research question is clearly motivated and the scope of the manuscript is “just right”. The authors also acknowledge potential limitations of the study in a transparent manner and are able to provide policy recommendations based on the results. Manuscripts of this level of quality are rarely submitted for review, and I am therefore pleased with the overall quality of the paper.

Below, I list a few minor comments and a few suggestions that the authors may wish to consider in order to further improve the manuscript. All comments provided are entirely optional.

Abstract and Introduction

In the introduction the research is clearly motivated, and I am particularly pleased with the clarity of the abstract.

Data and Methods

The data and methods are clearly described. The use of a GLM with a log link is appropriate and well justified. I consider it a particular strength that potential reverse causality is addressed by using a lagged variable.

Although information on how demand for ECEC is calculated is provided in the referred article, I suggest clarifying this further in the main text. Specifically, on page 7, line 151, it might be helpful to explain more explicitly how the main explanatory variable is defined (and later point out that the explanatory variable referring to demand is actually use). Please be very explicit about whether the outcome variable represents use, i.e., the number of 0–2-year-old children participating in early childhood education divided by the total number of children aged 0–2 in the region.

Small variation in some countries

It may also be worth noting that in some countries the use of early childhood education and care services has developed more slowly than in others due to policy changes, particularly related to parental leave schemes and home care allowance (cash-for-care benefit). This may be the case, for example, in the Czech Republic, Finland, possibly Hungary, as well as other Central European countries where parental leave durations have been extended (see, e.g., Bičáková & Kalíšsková, 2019; Canaan et al., 2022, Österbacka & Räsänen, 2022).

These parental leave or cash-for-care policies have, to some extent, reduced the use of early childhood education among the youngest children, while use has increased among slightly older age groups (e.g., lower participation among 0–1-year-olds and increased participation among one- and two-year-olds and older children). This point could be briefly mentioned, for instance in the discussion section or on page 9, line 199 (with a maximum of 1 or 2 sentences, since this issues is outside the scope of the present research).

Results

The results are clearly presented using figures, which I find effective and appropriate. In the first row of Figure 3 (first childbirth), the result for unemployed individuals appears somewhat puzzling, as the average marginal effect seems to decline as lagged regional day care use increases. However, as I understand it, the coefficients at value –10 and value +10 are proximately the same and the difference between coefficients from -10 to +10 are statistically insignificant. This suggests that among individuals in a weaker labour market attachment, the expansion of early childhood education services is not associated with fertility outcomes. This is also an interesting finding in itself.

Robustness

I appreciate that the authors have carefully considered issues arising from the EU-SILC panel structure and have tested alternative models and methods.

Conclusion

I particularly appreciate the inclusion of policy recommendations. The main message—that expanding the availability of early childhood education services, especially in regions where access is currently limited, may support family formation and possibly improve work–life balance and promote female employment—is well articulated and policy relevant.

References

Canaan, S., Lassen, A. S., Rosenbaum, P., & Steingrimsdottir, H. (2022). Maternity Leave and Paternity Leave: Evidence on the Economic Impact of Legislative Changes in High-Income Countries. In Oxford Research Encyclopedia of Economics and Finance. https://doi.org/10.1093/acrefore/9780190625979.013.806

Bičáková, A., & Kalíšsková, K. (2019). (Un)intended effects of parental leave policies: Evidence from the Czech Republic. Labour Economics. https://doi.org/10.1016/j.labeco.2019.07.003

Österbacka, E., & Räsänen, T. (2022). Back to work or stay at home? Family policies and maternal employment in Finland. Journal of Population Economics, 35(3), 1071–1101. https://doi.org/10.1007/s00148-021-00843-4

**********

6. PLOS authors have the option to publish the peer review history of their article (what does this mean?). If published, this will include your full peer review and any attached files.

If you choose “no”, your identity will remain anonymous but your review may still be made public.

**Do you want your identity to be public for this peer review?**  For information about this choice, including consent withdrawal, please see our Privacy Policy.

Reviewer #1: No

Reviewer #2: **Yes:** Tapio Räsänen

**********

[NOTE: If reviewer comments were submitted as an attachment file, they will be attached to this email and accessible via the submission site. Please log into your account, locate the manuscript record, and check for the action link "View Attachments". If this link does not appear, there are no attachment files.]

To ensure your figures meet our technical requirements, please review our figure guidelines: https://journals.plos.org/plosone/s/figures

You may also use PLOS’s free figure tool, NAAS, to help you prepare publication quality figures: https://journals.plos.org/plosone/s/figures#loc-tools-for-figure-preparation.

NAAS will assess whether your figures meet our technical requirements by comparing each figure against our figure specifications.

Attachment Submitted filename: Review comments.docx

# Author comment: Author response to Decision Letter 1

**DOI:** 10.1371/journal.pone.0353502.r002

8 Jun 2026

Dear Editor and Reviewers,

We are sincerely grateful for the reviewers’ constructive comments and insightful suggestions and the opportunity to represent this paper. In light of these comments, we have carefully revised the submitted manuscript. First, we have more explicitly acknowledged the limitations of ECEC usage as a proxy measure in the Data sub-section and the Limitations paragraph. This addresses a concern raised by both reviewers regarding the operationalization and interpretation of the main explanatory variable. Second, we have translated the initially presented relative associations into absolute transition rates to aid policy interpretation. Third, we have clarified the scope of the regional fixed-effects strategy, explicitly acknowledging that region and year dummies cannot account for region-specific time-varying confounders. Fourth, we have reframed the 22% ECEC threshold as an indicative, sample-specific benchmark rather than a universal policy target. In addition, all minor points raised by both reviewers have been considered and addressed, with corresponding edits made throughout the manuscript. Line numbers refer to the revised version.

Response to Reviewer 1’s comments

We sincerely thank Reviewer 1 for the thoughtful and constructive comments on our manuscript. Based on these comments, we have carefully revised the original manuscript. Please find below our responses and revisions.

Comment 1: Using ECEC as a Proxy

When childcare usage is used as a proxy for availability, policy supply is confused with eligibility requirements, prevailing norms, and parental demand. The estimated associations may reflect broader behavioral or labor-market changes rather than just childcare capacity, and this should be more clearly acknowledged in the manuscript.

Yes. We agree that the association between childcare usage and fertility might reflect not only change in availability or eligibility requirements (already noted in the discussion) but also in the demand side through, e.g., changes in parental norms and in the labor market. In the revised manuscript, the limitations of this proxy measure are now more explicitly acknowledged in the Data sub-section (line 154) and in the Discussion sections when presenting the study’s limitations (line 410). The corresponding changes in the revised main text are as follows:

Line 154: “[…] Actual enrolment, however, also serves as a proxy for availability given the general undersupply of public early childcare across European regions."

Lines 406: “Moreover, childcare usage might reflect not only the supply-side capacity and eligibility rules, but also a combination of prevailing gender norms around infant childcare enrolment, labor market changes, and parental preferences, meaning that the estimated associations may partly capture broader institutional and societal changes rather than childcare availability alone.”

Comment 2: Confounding that Changes Over Time

Although regional demeaning accounts for stable regional differences, it is unable to take into consideration time-varying unobserved factors, such as concurrent changes in family policy or changing employment conditions. This restriction calls for a more thorough explanation.

Yes. Our models include region-of-residence dummies and year-of-entry dummies that, together, absorb regional heterogeneity and account for common trends. The two-year lag on ECEC further reduces reverse causality concerns. However, region-specific time-varying unobserved factors, such as concurrent regional changes in the labor market or regional family policies other than ECEC, cannot be ruled out with the current design. Most policy changes, such as leaves or benefits, take place on the national level, though, that we can account for. We have clarified, in the Materials and Methods section, that region dummies and year dummies account for time-stable regional heterogeneity and a common time trend, separately, and explicitly acknowledged that our design cannot rule out region-specific time-varying unobserved factors, as a limitation. Specifically, we edited the manuscript as follows:

Line 186: “Region-of-residence dummies absorb time-stable differences across regions in fertility behavior, labor market structure, and family policy context, while year-of-entry dummies account for common temporal trends affecting all regions simultaneously. Together, they reduce concerns about confounding by stable compositional differences and aggregate period shocks. However, this strategy cannot account for region-specific time-varying unobserved factors, such as concurrent regional changes in labor market conditions or family support policies other than ECEC expansion.”

Line 410: “Additionally, although region and year dummies absorb time-stable regional heterogeneity and a common time trend, they cannot account for region-specific time-varying unobserved confounders, such as regional shifts in labor market conditions, parental leave provision, or other family policies.”

Comment 3: Short Panel and Interpretation of Fertility

Inference is limited to short-term parity transitions by the four-year EU-SILC panel. It is best to frame claims about a decrease in the difference between intended and actual fertility as suggestive rather than definitive.

We agree and thank the reviewer for raising this important comment. The four-year EU-SILC panel limits inference to short-term parity transitions, and effects on fertility should be framed as suggestive rather than definitive. We have revised the manuscript accordingly in the Abstract and the Discussion sections, as follows:

Line 26: "Our results suggest that investing in public childcare is associated, in the short term, with reducing the gap between intended and actual fertility in Europe, particularly in contexts where provision is lower.

Line 373: “What is more, childcare expansions might help in reducing the mismatch between desired and actual fertility, as indicated by higher short-term parity transition rates. Whether these associations persist and translate into higher completed fertility calls for future research employing longer follow-up windows.”

Lines 393: "Second, the short panel framework provided by EU-SILC comes with drawbacks stemming from left and right censoring, limiting our analysis to short-term parity transitions."

Comment 4: Absolute versus Relative Effects

Given the low baseline transition rates, large relative effects translate into modest absolute changes. More focus on absolute effect sizes would be beneficial for the policy debate in particular

We sincerely appreciate this suggestion. Focusing on absolute changes in transition rates is indeed more useful for the current policy debate, while acknowledging that baseline transition rates are small over the four-year panel. We have then translated relative associations in absolute increases in the Results and Discussion sections. The corresponding changes in the revised main text are as follows:

Line 246: “This translates to an increase in transition rates from 9.8 to 12.2 first births per 100 women and from 7.1 to 9.0 second births.”

Line 369: “[…] according to our findings, an increase of 10% in the share of children in public childcare is associated with an increase in first-birth transition rates from 9.8 to 12.2 births per 100 women, and from 7.1 to 9.0 second births, in the following four years – equivalent to about a one-third increase in transition rates.”

Comment 5: Effects of Saturation

A significant contribution is the identification of thresholds beyond which ECEC expansion exhibits no correlation with fertility; however, these cut-points are model-dependent and empirically derived. They shouldn't be offered as exact standards for policy.

We agree. The ECEC threshold of 22%, above which we find no association between ECEC increase and fertility is model and data dependent. The empirical distributional values (quartiles) calculated on the administrative childcare data itself, are presented in lines 292–294 of the original manuscript: "[…] 25% of regions in our sample reporting an overall average ECEC below 6%, while 50% are below 12%, and 75% are below 23%." Therefore, our findings show that the association between ECEC expansion and fertility is significant below this quartile boundary (i.e., in the bottom three quarters of the European regional childcare distribution available in our analytical sample) and weakens in the top quartile where childcare is already relatively common. Yet, this threshold needs to be interpreted as an indicative, sample-specific benchmark rather than a universal policy standard, given that the analytical sample does not cover all EU regions and that existing policy targets are informed by broader criteria beyond the childcare-fertility association alone. We have then redacted the manuscript as follows:

Line 304: "In regions where public ECEC exceeds 22% overall (i.e., 75% of the regions in our sample), we find no statistically significant association […]."

Line 363: "These thresholds however reflect the distributional structure of our analytical sample and should be interpreted as indicative benchmarks rather than universal policy standards, given that existing policy targets are informed by broader criteria beyond the childcare-fertility association alone."

Line 426: “[…] especially in regions where said services are insufficient (i.e., three quarters of the regions in our data, that show an average childcare usage below 23% and have not departed from such threshold by 2019) […]."

Small Points

The Own-Children Approach

Although the robustness checks are comforting, transparency would be enhanced by a brief statement recognizing that residual misclassification may differ depending on socioeconomic status.

We have edited the manuscript to acknowledge the reviewer’s point in the limitations paragraph. While some methodological studies on the Own-Children Approach find no major systematic differences between social groups in the underestimation of fertility (Greulich and Dasré 2017; Krapf and Kreyenfeld 2015), other find, using French data, that the number of children living outside the household is not randomly distributed, pointing to socioeconomic differentials and a systematic bias in the measurement, where underestimation particularly affects lower-educated women and those with a migration background (Greulich and Dasré 2019).

Line 391: “Although misclassification bias in the own-children method is unlikely to bias fertility estimates substantially [32,51], residual misclassification might be unevenly distributed across socioeconomic groups [31].”

31. Greulich, Angela, and Aurélien Dasré. 2019. ‘Observing the Number of Children with EU-SILC: A Quantification of Biases’. Population Vol. 73(4):685–720. doi:10.3917/popu.1804.0719.

32. Greulich, Angela, and Aurélien Dasré. 2017. ‘The Quality of Periodic Fertility Measures in EU-SILC’. Demographic Research 36:525–56. doi:10.4054/DemRes.2017.36.17.

51. Krapf, Sandra, and Michaela R. Kreyenfeld. 2015. Fertility Assessment with the Own-Children Method: A Validation with Data from the German Mikrozensus. 0 edn. TR-2015-003. Rostock: Max Planck Institute for Demographic Research. doi:10.4054/MPIDR-TR-2015-003.

Selecting a Country

In addition to the assurance provided by the national-level robustness checks, the exclusion of nations without sub-national data should be more prominently acknowledged.

We have added a sentence explicitly acknowledging the exclusion of some countries due to the missing information on the sub-national region of residence in the Materials and methods section. Precisely, in line 128 we reviewed the manuscript as follows: “[…] and excluded countries lacking sub-national residence data in EU-SILC, which are listed in Supporting Information, Table S1. Sensitivity analyses confirm that including these countries using national-level childcare measures does not alter our main findings.”

The Impact of Anticipation

Although anticipation effects might not be captured, lagging ECEC lowers the possibility of reverse causality. The methodological discussion would be strengthened by a brief recognition of this possibility.

We acknowledge that lagging ECEC by two years, while helping to partially rule out reverse causality, may not capture anticipation effects. Therefore, we have updated the manuscript as follows:

Line 162: “To reduce the likelihood of reverse causality, ECEC is lagged by two years, although this does not, in principle, preclude anticipation effects, whereby couples may adjust fertility intentions in response to announced childcare expansions in later years.”

Causal Terminology

To ensure consistency with the associational framework used throughout, the discussion should be examined for any language that might be interpreted as suggesting causation.

We have scanned the manuscript for any residual causal-sounding verbs or phrases that slip through despite the overall associational framing, as explicitly disclaimed in lines 357–360 in the submitted manuscript. Precisely, we have revised the manuscript as follows:

Line 237: “One percentage point increase in ECEC is associated with an increase in first-birth rates of 0.24 points (AME=0.0024, P=0.001) and in second-birth rates of 0.19 points (AME=0.0019, P<0.001).”

Line 333: “These factors are likely pathways underlying the reported positive associations, which have been found to promote total fertility and reduce postponement”

Line 422: “Increasing the provision of early child education and care services instead not only invest explicitly in the future generation but is also associated with greater gender equality, which in turn has been linked with higher fertility.”

Line 425: “Overall, our results provide a clear picture of the role of publicly subsidized childcare in supporting transitions to parenthood and to second births, especially in regions where said services are insufficient […]”

Response to Reviewer 2’s comments

We sincerely thank Reviewer 2 for the kind words and the valuable suggestions concerning our manuscript. In response to these suggestions, we have revised the manuscript accordingly, and our detailed responses to the comments that suggested a revision are presented below in blue.

Comment 1: Data and Methods

Although information on how demand for ECEC is calculated is provided in the referred article, I suggest clarifying this further in the main text. Specifically, on page 7, line 151, it might be helpful to explain more explicitly how the main explanatory variable is defined (and later point out that the explanatory variable referring to demand is actually use). Please be very explicit about whether the outcome variable represents use, i.e., the number of 0–2-year-old children participating in early childhood education divided by the total number of children aged 0–2 in the region.

We agree and thank the reviewer for pointing this out. We made explicit the precise operationalization of our main variable of interest (ECEC) directly in the text rather than deferred to Scherer & Pavolini (2023). We then updated the manuscript as follows:

Line 150: “The main explanatory variable is the yearly regional rate of public early childcare (ECEC) usage for children aged 0–2, demeaned at the regional level and lagged by two years. This is defined as the number of children aged 0–2 enrolled in publicly subsidized childcare divided by the total number of children aged 0–2 in the region, and captures actual enrolment rather than the number of available slots. Actual enrolment, however, also serves as a proxy for availability given the general undersupply of public early childcare across European regions.”

Comment 2: Small variation in some countries

It may also be worth noting that in some countries the use of early childhood education and care services has developed more slowly than in others due to policy changes, particularly related to parental leave schemes and home care allowance (cash-for-care benefit). This may be the case, for example, in the Czech Republic, Finland, possibly Hungary, as well as other Central European countries where parental leave durat

Attachment Submitted filename: Response to Reviewers.docx

# Peer review: Decision Letter 1

**DOI:** 10.1371/journal.pone.0353502.r003

**Authors:** Iacopo Odoardi, Iacopo Odoardi

24 Jun 2026

Early public childcare and fertility: A longitudinal study for Europe

PONE-D-26-07298R1

Dear Dr. Minchio,

We’re pleased to inform you that your manuscript has been judged scientifically suitable for publication and will be formally accepted for publication once it meets all outstanding technical requirements.

Within one week, you’ll receive an e-mail detailing the required amendments. When these have been addressed, you’ll receive a formal acceptance letter and your manuscript will be scheduled for publication.

An invoice will be generated when your article is formally accepted. Please note, if your institution has a publishing partnership with PLOS and your article meets the relevant criteria, all or part of your publication costs will be covered. Please make sure your user information is up-to-date by logging into Editorial Manager at Editorial Manager® and clicking the ‘Update My Information' link at the top of the page. For questions related to billing, please contact billing support.

If your institution or institutions have a press office, please notify them about your upcoming paper to help maximize its impact. If they’ll be preparing press materials, please inform our press team as soon as possible -- no later than 48 hours after receiving the formal acceptance. Your manuscript will remain under strict press embargo until 2 pm Eastern Time on the date of publication. For more information, please contact onepress@plos.org.

Kind regards,

Iacopo Odoardi, Ph.D.

Academic Editor

PLOS One

Additional Editor Comments:

Dear Authors, both reviewers have expressed their satisfaction with the revisions and acknowledge that their comments have been appropriately addressed. They also appreciate the improvements made to the manuscript following the review process. In light of the positive evaluations received, I am pleased to accept your manuscript for publication.

Reviewers' comments:

Reviewer's Responses to Questions

**Comments to the Author**

1. If the authors have adequately addressed your comments raised in a previous round of review and you feel that this manuscript is now acceptable for publication, you may indicate that here to bypass the “Comments to the Author” section, enter your conflict of interest statement in the “Confidential to Editor” section, and submit your "Accept" recommendation.

Reviewer #1: All comments have been addressed

Reviewer #2: All comments have been addressed

**********

2. Is the manuscript technically sound, and do the data support the conclusions?

The manuscript must describe a technically sound piece of scientific research with data that supports the conclusions. Experiments must have been conducted rigorously, with appropriate controls, replication, and sample sizes. The conclusions must be drawn appropriately based on the data presented.

Reviewer #1: Yes

Reviewer #2: Yes

**********

3. Has the statistical analysis been performed appropriately and rigorously?

Reviewer #1: Yes

Reviewer #2: Yes

**********

4. Have the authors made all data underlying the findings in their manuscript fully available?

The PLOS Data policy requires authors to make all data underlying the findings described in their manuscript fully available without restriction, with rare exception (please refer to the Data Availability Statement in the manuscript PDF file). The data should be provided as part of the manuscript or its supporting information, or deposited to a public repository. For example, in addition to summary statistics, the data points behind means, medians and variance measures should be available. If there are restrictions on publicly sharing data—e.g. participant privacy or use of data from a third party—those must be specified.

Reviewer #1: (No Response)

Reviewer #2: Yes

**********

5. Is the manuscript presented in an intelligible fashion and written in standard English?

PLOS ONE does not copyedit accepted manuscripts, so the language in submitted articles must be clear, correct, and unambiguous. Any typographical or grammatical errors should be corrected at revision, so please note any specific errors here.

Reviewer #1: Yes

Reviewer #2: Yes

**********

6. Review Comments to the Author

Please use the space provided to explain your answers to the questions above. You may also include additional comments for the author, including concerns about dual publication, research ethics, or publication ethics. (Please upload your review as an attachment if it exceeds 20,000 characters)

Reviewer #1: All the comments have been addressed properly. I am happy with the revision. The paper will be a good contribution to the knowledge.

Reviewer #2: Thank you for addressing all of the minor comments and suggestions. The manuscript is excellent and, in my view, suitable for publication.

**********

7. PLOS authors have the option to publish the peer review history of their article (what does this mean?). If published, this will include your full peer review and any attached files.

If you choose “no”, your identity will remain anonymous but your review may still be made public.

**Do you want your identity to be public for this peer review?**  For information about this choice, including consent withdrawal, please see our Privacy Policy.

Reviewer #1: No

Reviewer #2: **Yes:** Tapio Räsänen

**********

# Editor report: Acceptance letter

**DOI:** 10.1371/journal.pone.0353502.r004

**Authors:** Iacopo Odoardi, Iacopo Odoardi

PONE-D-26-07298R1

PLOS One

Dear Dr. Minchio,

I'm pleased to inform you that your manuscript has been deemed suitable for publication in PLOS One. Congratulations! Your manuscript is now being handed over to our production team.

At this stage, our production department will prepare your paper for publication. This includes ensuring the following:

* All references, tables, and figures are properly cited

* All relevant supporting information is included in the manuscript submission,

* There are no issues that prevent the paper from being properly typeset

You will receive further instructions from the production team, including instructions on how to review your proof when it is ready. Please keep in mind that we are working through a large volume of accepted articles, so please give us a few days to review your paper and let you know the next and final steps.

Lastly, if your institution or institutions have a press office, please let them know about your upcoming paper now to help maximize its impact. If they'll be preparing press materials, please inform our press team within the next 48 hours. Your manuscript will remain under strict press embargo until 2 pm Eastern Time on the date of publication. For more information, please contact onepress@plos.org.

You will receive an invoice from PLOS for your publication fee after your manuscript has reached the completed accept phase. If you receive an email requesting payment before acceptance or for any other service, this may be a phishing scheme. Learn how to identify phishing emails and protect your accounts at https://explore.plos.org/phishing.

If we can help with anything else, please email us at customercare@plos.org.

Thank you for submitting your work to PLOS ONE and supporting open access.

Kind regards,

PLOS ONE Editorial Office Staff

on behalf of

Prof. Iacopo Odoardi

Academic Editor

PLOS One
